# Magnetic anisotropy of endohedral lanthanide ions: paramagnetic NMR study of MSc_2_N@C_80_-*I*_h_ with M running through the whole 4f row†
†Electronic supplementary information (ESI) available: Details of relaxation time measurements, ligand field splitting, additional correlation between data points, Cartesian coordinates. See DOI: 10.1039/c5sc00154d



**DOI:** 10.1039/c5sc00154d

**Published:** 2015-01-28

**Authors:** Y. Zhang, D. Krylov, M. Rosenkranz, S. Schiemenz, A. A. Popov

**Affiliations:** a Leibniz Institute for Solid State and Materials Research , 01069 Dresden , Germany . Email: a.popov@ifw-dresden.de

## Abstract

Paramagnetic and variable temperature ^13^C and ^45^Sc nuclear magnetic resonance studies are performed for nitride clusterfullerenes MSc_2_N@C_80_ with icosahedral *I*_h_(7) carbon cage, where M runs through all lanthanides forming nitride clusters. The influence of the endohedral lanthanide ions on the NMR spectral pattern is carefully followed, and dramatic differences are found in peak positions and line widths. Thus, ^13^C lines broaden from 0.01–0.02 ppm in diamagnetic MSc_2_N@C_80_ molecules (M = La, Y, Lu) to several ppm in TbSc_2_N@C_80_ and DySc_2_N@C_80_. Direction of the paramagnetic shift depends on the shape of the 4f electron density in corresponding lanthanide ions. In TmSc_2_N@C_80_ and ErSc_2_N@C_80_ with prolate 4f-density of lanthanide ions, ^13^C signals are shifted down-field, whereas ^45^Sc peaks are shifted up-field *versus* diamagnetic values. In all other MSc_2_N@C_80_ molecules lanthanide ions have oblate-shaped 4f electron density, and the lanthanide-induced shift is negative for ^13^C and positive for ^45^Sc peaks. Analysis of the pseudocontact and contact contributions to chemical shifts revealed that the pseudocontact term dominates both in ^13^C and ^45^Sc NMR spectra, although contact shifts for ^13^C signals are also considerable. Point charge computations of the ligand field splitting are performed to explain experimental results, and showed reasonable agreement with experimental pseudocontact shifts. Nitrogen atom bearing large negative charge and located close to the lanthanide ion results in large magnetic anisotropy of lanthanide ions in nitride clusterfullerenes with quasi-uniaxial ligand field.

## Abstract 2

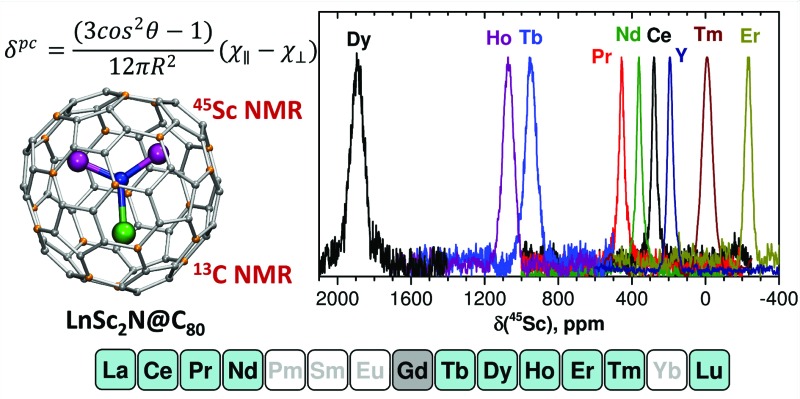
Paramagnetic and variable temperature ^13^C and ^45^Sc nuclear magnetic resonance studies are performed for nitride clusterfullerenes MSc_2_N@C_80_ with icosahedral *I*_h_(7) carbon cage, where M runs through all lanthanides forming nitride clusters.

## Introduction

The molecules of endohedral metallofullerenes (EMFs) comprise the fullerene cage encapsulating one to three metal ions or a more complex hybrid cluster.^1^ Shielding magnetic ions (such as lanthanides) inside the carbon cage protects their spin states from the environment making EMFs potentially useful for spintronics applications. Numerous studies of magnetic properties of EMFs reported before 2012 did not reveal any deviation from paramagnetic behaviour, although the influence of the magnetic anisotropy of endohedral lanthanide ions on the measured magnetic moments was proposed.^2^ However, in 2012 it was discovered that DySc_2_N@C_80_ exhibits slow magnetization reversal at helium temperatures.^3^ The presence of hysteresis in the magnetization curves measured below 5 K indicated that this EMF can be classified as a single molecule magnet (SMM). In subsequent studies, the SMM behaviour was also found in Dy_2_ScN@C_80_ (ref. 4) and HoSc_2_N@C_80_,^5^ and more EMF-based SMM are likely to be discovered in the near future. Importantly, both DySc_2_N@C_80_ and Dy_2_ScN@C_80_ exhibit unusually long attempt times, which are orders of magnitude longer than in other lanthanide-based SMMs.

One of the most important factors determining the SMM behaviour of lanthanide molecular magnets is the single ion magnetic anisotropy.^6^ Reliable elucidation of the ligand-field (LF) splitting of the 4f^*n*^ total momentum states is crucial (although not sufficient) for the correct description of magnetic properties of lanthanide SMMs. Despite the understanding that the magnetic anisotropy is an important factor determining magnetic properties of EMF, a consistent description of the LF effects in EMF has not been provided yet. The first dedicated studies of the LF in nitride clusterfullerenes have been published only recently and were motivated by the discovery of SMM behaviour in DySc_2_N@C_80_ and Dy_2_ScN@C_80_. According to the *ab initio* computations at the CASSCF level,^7^ the LF splitting in DySc_2_N@C_80_ is so large that its magnetization behavior at low temperature is determined solely by the ground state of the ^6^H_15/2_ manifold. Namely, the gap between the ground (*m*_J_ = ±15/2) and the first excited (*m*_J_ = ±13/2) magnetic states was predicted to be 373–415 cm^–1^ (ref. 7a) or 485 cm^–1^ (ref. 7b). Likewise, magnetization curves of DySc_2_N@C_80_ and HoSc_2_N@C_80_ measured by SQUID below 10 K are also described by only one state with *m*_J_ = ±15/2 (Dy) or *m*_J_ = ±8 (Ho).^4,5^ The information about excited magnetic states might be revealed from magnetizations studies at higher temperatures (*e.g.*, the population of the *m*_J_ = ±13/2 state in DySc_2_N@C_80_ near room temperature is expected to be *ca.* 10%). However, with the increase of the temperature the total magnetization of the sample is decreasing dramatically and can hardly be measured reliably when only limited amount of sample is available (as it is often the case with EMF SMMs). An alternative might be the use of spectroscopic techniques, such as analysis of the fine structure in the lanthanide-based luminescence spectrum.^8^ Unfortunately, the fullerene cages of EMFs absorb light in the visible range and hence block the possibility to observe the lanthanide-based luminescence for a majority of 4f elements. So far, metal-based luminescence in EMFs could be detected only for endohedral Er^3+^ ions emitting in the near-IR range.^9^

Solution nuclear magnetic resonance (NMR) studies can provide complimentary information on the magnetic anisotropy of lanthanide ions for the temperatures not well accessible by direct magnetization measurements. The chemical shift of a nuclei in a paramagnetic compound can be described as a sum of diamagnetic and paramagnetic terms, *δ* ^exp^ = *δ* ^dia^ + *δ* ^para^. In due turn, the paramagnetic shift has two major contributions, contact *δ* ^con^ and pseudocontact *δ* ^pc^.^10^ The contact (Fermi) shift results from the interaction between the nuclear spin (of ^13^C or ^45^Sc atoms in this work) and the spin-polarized electron density of the molecule (in particular in the region close to the nuclei of interest). As such, the contact shift is proportional to the hyperfine coupling constant weighed with the expectation value of the spin operator *S*_*z*_ of the lanthanide. The pseudocontact shift is caused by dipolar through-space interactions of the nuclear and electronic magnetic dipoles. For an *i*-th atom in a lanthanide-containing molecule the pseudocontact shift can be computed as:1a
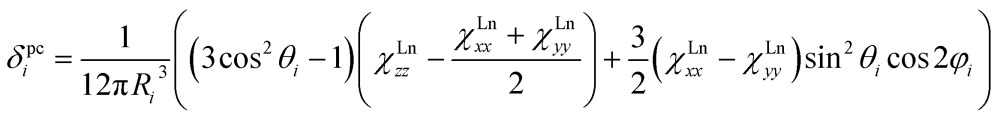
where *χ*Ln*αα* are components of the magnetic susceptibility tensor of the lanthanide, whereas *R*_*i*_, *θ*_*i*_, and *φ*_*i*_ are polar coordinates of the *i*-th atom in the coordinate system centred on the lanthanide ion. In particular, *R*_*i*_ is a distance between the atom of interest and lanthanide ion, and *θ*_*i*_ is an angle between quantization axis *z* and the vector connecting the lanthanide ion and the *i*-th atom. If *χ*Ln*xx* = *χ*Ln*yy* (*i.e.* the ligand field is uniaxial), eqn (1a) is simplified to:1b
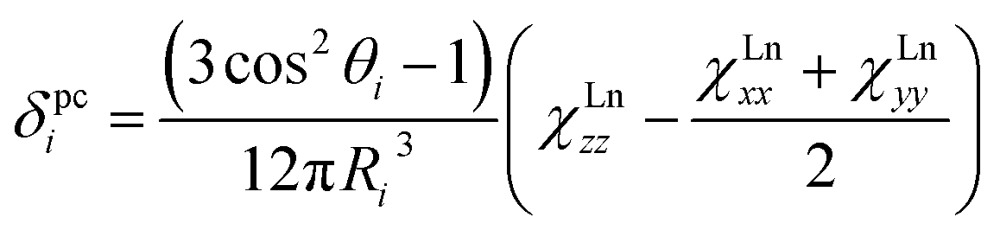



As can be seen in eqn (1), the pseudocontact shift contains information on the molecular structure and on the magnetic properties of the paramagnetic center. Paramagnetic NMR is therefore a popular structure elucidation tool for metal complexes, polymers or biomolecules.^10b,c,11^ At the same time, it can be also used to determine or verify LF parameters in lanthanide complexes.^12^ The prerequisite for both types of application of NMR spectroscopy in the studies of paramagnetic molecules is the possibility to separate contact and pseudocontact contributions,^13^ since only the latter brings necessary information about molecular structure and magnetic anisotropy.

In the field of endohedral metallofullerenes, paramagnetic NMR was mainly used for the structural studies. Several Ce-EMFs,^14^ anion of Pr@C_82_,^15^ three isomers of Tm@C_82_,^16^ Sm@C_80_,^17^ and Sm@C_82_ (ref. 18) were characterized by ^13^C NMR. The variable-temperature ^45^Sc NMR study was also reported for CeSc_2_N@C_80_.^19^ The dominant contribution of the pseudocontact shift in Ce-EMFs was postulated based on the analysis of the temperature dependence in the framework of Bleaney's theory. The size of the contact term contribution in the paramagnetic NMR spectra of EMFs remains unclear. Our group recently reported paramagnetic NMR study of HoM_2_N@C_80_ and Ho_2_MN@C_80_ (M = Sc, Y, Lu).^20^ ^13^C and ^45^Sc signals of these molecules could be identified in spite of the severe broadening induced by Ho^3+^ ions. This study showed that paramagnetic NMR spectroscopy when combined with the analysis of the ligand field splitting, can provide information on the magnetic state of lanthanide ions in EMFs.

In this work we report on a systematic paramagnetic NMR study of MSc_2_N@C_80_ compounds with all lanthanides forming nitride clusterfullerenes (M = La, Ce, Pr, Nd, Gd, Tb, Dy, Ho, Er, Tm) and concomitant calculations of the ligand field splitting in these molecules. The main goal is to provide a consistent and uniform description of the magnetic states of lanthanide ions in these molecules based on the ligand field computations verified by experimental NMR data. The manuscript is organized in several parts. First, we describe the results of experimental NMR measurements, including the temperature dependence and relaxation times. Then, we perform a thorough analysis of the NMR data to separate contact and pseudocontact contributions to paramagnetic shifts. This section is followed by the brief analysis of the molecular structures. Then point-charge calculations of the ligand field splitting are described, which give the desired description of the single ion magnetic anisotropy in the series of MSc_2_N@C_80_ molecules across the whole 4f row. Reliability of the computed LF patterns is then verified by comparison of the computed and experimental pseudocontact shifts.

## Experimental and computational details

**Fig. 1:**
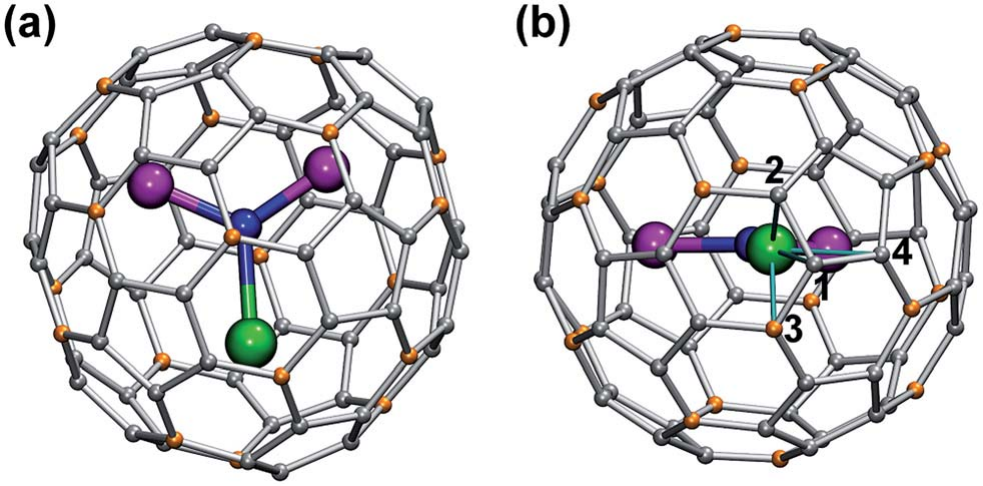
DFT-optimized molecular structure of representative MSc_2_N@C_80_-*I*_h_ (**1Pr**). Praseodymium ion (shown green) is at the distance of 2.225 Å from the central nitrogen atom (blue), whereas two Sc–N distances are 1.939 and 1.936 Å. Carbon atoms are shown in grey (pentagon–hexagon–hexagon junctions, PHHJs) and orange (triple hexagon junctions, THJs). In (b), the plane of the cluster is normal to the paper. Thin cyan lines denote shortest Pr–C distances: 2.508 Å (C1), 2.553 Å (C2), 2.591 Å (C3), and 2.658 Å (C4).

In this work we will consider exclusively MSc_2_N@C_80_ compounds with the *I*_h_(7) carbon cage (Fig. 1). Hereafter the compounds will be abbreviated as **1M**, where M denotes the corresponding lanthanide (*e.g.*, LaSc_2_N@C_80_ is denoted hereafter as **1La**). All studied compounds were synthesized using the arc-discharge method in the presence of nitrogen source such as NH_3_ (ref. 21) or guanidine thiocyanate,^22^ and then separated using chromatography as described in the ESI Fig. S1–S5.† The synthesis of **1Ce**,^23^ **1Nd**,^24^ **1Pr**,^23^ **1Dy**,^24^ **1Ho**,^20*b*^ and **1Lu** ^24^ in our group was described earlier. **1La**, **1Pr**, **1Tb**, **1Er** and **1Tm** were synthesized for this work using guanidine thiocyanate as a nitrogen source, whereas **1Tb** and **1Y** were synthesized using NH_3_ as a reactive gas. Note that the synthesis and characterization of **1Y**,^25^ **1La**,^26^ **1Ce**,^19^ **1Tb**,^27^ and **1Er** ^28^ was also reported by other groups using somewhat different conditions. Gd also forms mixed-metal nitride clusterfullerenes with Sc,^29^ but neither ^13^C nor ^45^Sc NMR signals could be detected for **1Gd**, so this molecule will not be discussed hereafter.

The 125 MHz ^13^C NMR and 121.5 MHz ^45^Sc NMR measurements were performed on a Bruker Avance 500 spectrometer equipped with a multiprobe head 1152Z. The measurements were performed for compounds dissolved in CS_2_ with d^6^-acetone placed in a coaxial tube as a lock; in special cases d^4^-*ortho*-dichlorobenzene was also used as a solvent. Typically the spectra were measured at each 10 K in the temperature rage form 268 to 308 K.

Longitudinal relaxation rates (*R*_1_) were measured using inversion recovery pulse sequence with different delays between π and π/2 pulses. The values were determined from the slope of the linear fit of ln(*I*_max_ – *I*) *versus τ* (delay). Transversal relaxation rates (*R*_2_) were measured using Carr–Pucell–Meiboom–Gill (CPMG) pulse sequence.^30^ The values were determined from slopes of linear fit of –ln(*I*) *versus τ* (delay). In case of broad line width, *R*_2_ cannot be determined by CPMG-pulse sequence because the relaxation is too fast. Conservative estimation of *R*_2_ values in such cases can be done by measuring the line width at half peak maximum. However, R*2 determined by this procedure includes the line broadening by inhomogeneity of the magnetic field (
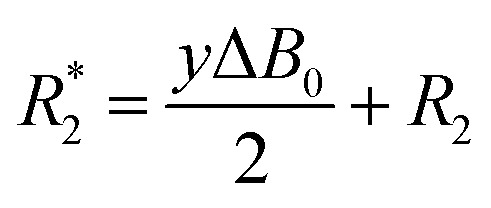
) and therefore the *T*_2_ time determined from the line width is shorter than the real one.

DFT optimization of molecular coordinates was performed at the B3LYP level using the Firefly^31^ code. The basis sets were def2-SVP {5,1,1/3,1/1} for carbon atoms,^32^ def2-TZVP {6,2,1,1/4,1,1/1,1/1} for nitrogen,^32^ and Stuttgart–Cologne effective core potentials for Sc (ECP10MDF)^33^ and lanthanides (4f-in-core ECP-MWB-II)^34^ with {3,1,1,1,1,1/2,2,1,1,1/4,1,1/1,1/1} and {3,1,1,1,1,1/3,1,1,1,1/2,1,1,1,1/1,1,1/1,1} valence parts, respectively. Spin density and hyperfine coupling constants in GdSc_2_N@C_80_ were computed at the PBE level using the specially tailored SARC basis set of TZVP quality^35^ combined with either DKH or ZORA scalar relativistic corrections implemented in the ORCA package.^36^ To compute the Bader (QTAIM) charges, full electron calculations were performed at the PBE/SARC-TZVP level with DKH scalar relativistic corrections using ORCA. To avoid poor reliability of the full electron DFT treatment of the 4f elements, Y was used to model the charges of the lanthanides from Tb to Tm, whereas La was used to model Ce, Pr, and Nd. QTAIM computations of the atomic charges were then performed with the AIMAll^37^ code. Point charge calculations of the ligand field splitting, magnetic susceptibility tensors, and 4f electron densities were performed using so1ion (Cfield) routine in McPhase code.^38^ Molecular structures and isosurfaces were visualized with the help of VMD.^39^

## Results and discussion

### NMR spectroscopy

#### ^13^C and ^45^Sc NMR spectra at 288 K

**Fig. 2:**
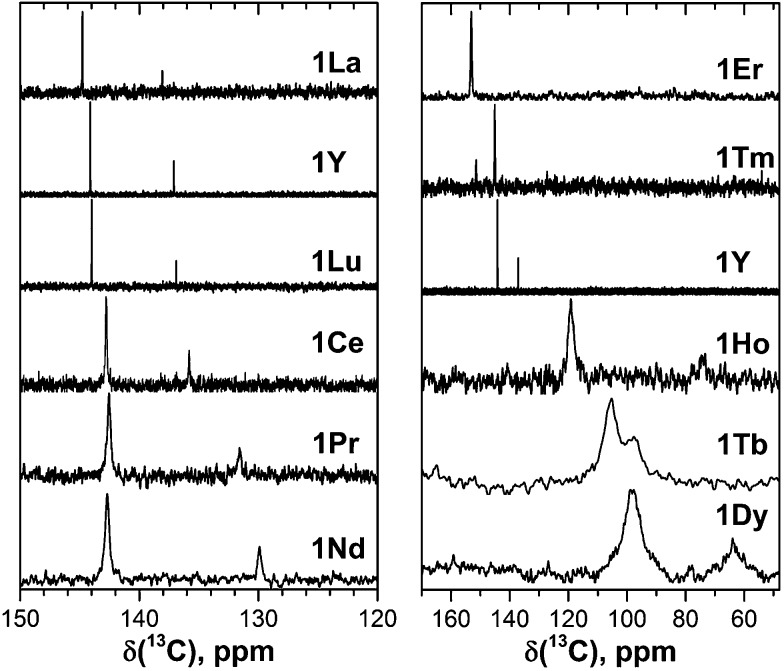
^13^C NMR spectra of **1M** compounds measured in CS_2_ at 288 K. Note the different scale in the left and right panels; for a better comparison, the spectrum of **1Y** is shown in both panels.

**Fig. 3:**
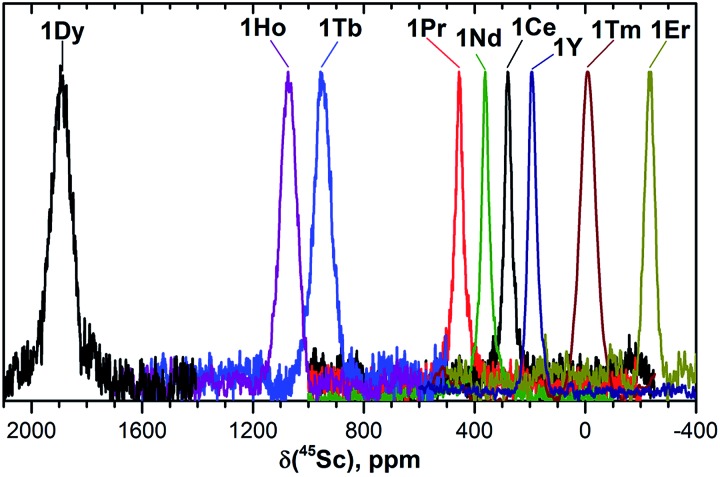
^45^Sc NMR spectra of **1M** compounds measured in CS_2_ at 288 K. The *δ*(^45^Sc) value of diamagnetic **1Y** is 191 ppm.

Fig. 2 and 3 show ^13^C and ^45^Sc NMR spectra of **1M** compounds measured at 288 K (the temperature is chosen as the center of the temperature interval used in the variable temperature NMR studies, see below).

Fast rotation of the MSc_2_N cluster inside the highly symmetric C_80_-*I*_h_ carbon cage results in the averaging of the carbon signals, leaving only two ^13^C NMR peaks with a 3 : 1 intensity ratio. The higher intensity peaks corresponds to the 60 carbon atoms at the pentagon–hexagon–hexagon junctions (PHHJ), whereas the lower intensity peak is due to the 20 atoms at the triple hexagon junctions (THJ, see Fig. 1). The chemical shift of the PHHJ carbons in diamagnetic **1M** compounds is bracketed by the **1La** and **1Lu** values, 144.76 and 143.99 ppm, respectively. Likewise, the THJ signal varies from 138.08 ppm in **1La** to 136.90 ppm in **1Lu**. Chemical shifts of the THJ and PHHJ carbon atoms in **1Y** are intermediate between those of **1La** and **1Lu** (see Table 1). Thus, depending on the size of the endohedral cluster, the diamagnetic ^13^C values are subject to change within the range of *ca.* 1 ppm (see also ref. 40) which should be taken into account in the analysis of the paramagnetic shifts in other **1M** compounds. The *δ* ^dia^ values listed in Table 1 and used to compute paramagnetic shifts were obtained by approximating the ^13^C chemical shifts by a quadratic function of the Shannon radii of metal ions (see ESI Fig. S6†).

**Table 1:** Shannon ionic radii of lanthanides and chemical shifts of **1M** molecules measured at 288 K^*a*^

**1M**	R(M^3+^)	^13^C-PHHJ		*δ* ^dia^ ^*b*^	*δ* ^para^	Δ308268	^13^C-THJ		*δ* ^para^	Δ308268	^45^Sc *δ*	*δ* ^para^ ^*c*^	Δ308268
		*δ*	Δ*ν*_1/2_				*δ*	*δ* ^dia^ ^*b*^					
Y	0.900	144.11	1.5	144.11	—	—	137.11	137.11	—	—	191	—	—
La	1.032	144.76	2.0	144.76	—	—	138.08	138.08	—	—	198	—	—
Ce	1.010	142.69	15.1	144.63	–1.94	0.73	135.73	137.89	–2.16	0.57	279	79	—
Pr	0.990	142.55	41.8	144.51	–1.96	0.72	131.59	137.73	–6.14	0.94	455	255	–43
Nd	0.983	142.69	50.5	144.47	–1.78	0.25	129.91	137.67	–7.76	1.48	361	161	–15
Tb	0.923	105.6	579	144.20	–38.6	8.6	98.0	137.25	–39.3	7.5	949	749	–174
Dy	0.912	97.5	905	144.15	–46.7	10.2	61.6	137.18	–75.6	—	1892	1692	–290
Ho	0.901	119.1	284	144.11	–25.0	—	74.2	137.12	–62.9	—	1072	872	–165
Er	0.890	153.23	∼45	144.08	9.15	–2.12	153.23	137.05	16.18	–3.12	–233	–433	109
Tm	0.880	144.83	17.2	144.04	0.79	0.34	151.97	137.00	14.97	–2.97	–10	–210	32
Lu	0.861	143.99	1.0	143.99	—		136.90	136.90	—		200	—	

^*a*^Ionic radii are in Å, chemical shifts are in ppm, line width Δ*ν*_1/2_ in Hz ^13^C chemical shifts are determined with precision to the second decimal except for **1Tb**, **1Dy**, and **1Ho**, which are determined to the first decimal.

^*b*^Diamagnetic shifts were estimated from the polynomial fit of the *δ*(**1M**)-*versus*-R(M^3+^) data for **1Y**, **1La**, and **1Lu**.

^*c*^The values are obtaining by subtracting the ^45^Sc chemical shift of **1La** (**1Ce**, **1Pr**, **1Nd**) or **1Lu** (other compounds).

The measurement of the ^13^C NMR spectra of paramagnetic EMFs is complicated by the considerable line broadening. However, thanks to the simple two-line spectrum of the C_80_ carbon cage, the signals are detectable even when they are severely broadened as in **1Tb** or **1Dy**. In **1Ce**, **1Pr**, **1Nd**, **1Tb**, **1Dy**, and **1Ho** the ^13^C signals are shifted up-field with respect to the diamagnetic values. The magnitude of the paramagnetic shift varies from few ppm in **1Ce**, **1Pr**, and **1Nd** to tens of ppm in **1Tb**, **1Dy**, and **1Ho** (Fig. 2 and Table 1). On average, lanthanide-induced shifts of the THJ carbons are more pronounced than those of the PHHJ carbons.

In **1Er** and **1Tm** the lanthanide-induce shift is positive (down-field). The PHHJ and THJ ^13^C signals of **1Er** are coinciding near the room temperature and appear at 153.2 ppm, but can be resolved at higher or lower temperatures as discussed below. In **1Tm**, the stronger lanthanide-induced shift of the THJ signal pushes it to a lower field than that of the PHHJ (in all other **1M** compounds the PHHJ signal appears at lower field than that of the THJ carbons). Direction of the lanthanide-induced paramagnetic shift is determined by a magnetic anisotropy of the lanthanide ion (see ref. 41 for recent examples), and hence experimental NMR data gives information at least on the sign of the anisotropy. This question will be discussed in more details below.

In ^45^Sc NMR spectra, all **1M** compounds exhibit one relatively broad peak (Fig. 3). The ^45^Sc chemical shifts of the diamagnetic **1La** and **1Lu** are 198 and 200 ppm, respectively, whereas in paramagnetic **1M** molecules the ^45^Sc signal position spans the range from –233 ppm in **1Er** to 1892 ppm in **1Dy**. The peak width varies from 30–40 ppm for the diamagnetic **1Lu** and **1La** and paramagnetic **1Ce**, **1Pr**, **1Nd** and **1Er**, to *ca.* 70–80 ppm for the **1Tb**, **1Dy**, **1Ho**, and **1Tm**.

#### Variable temperature NMR studies

**Fig. 4:**
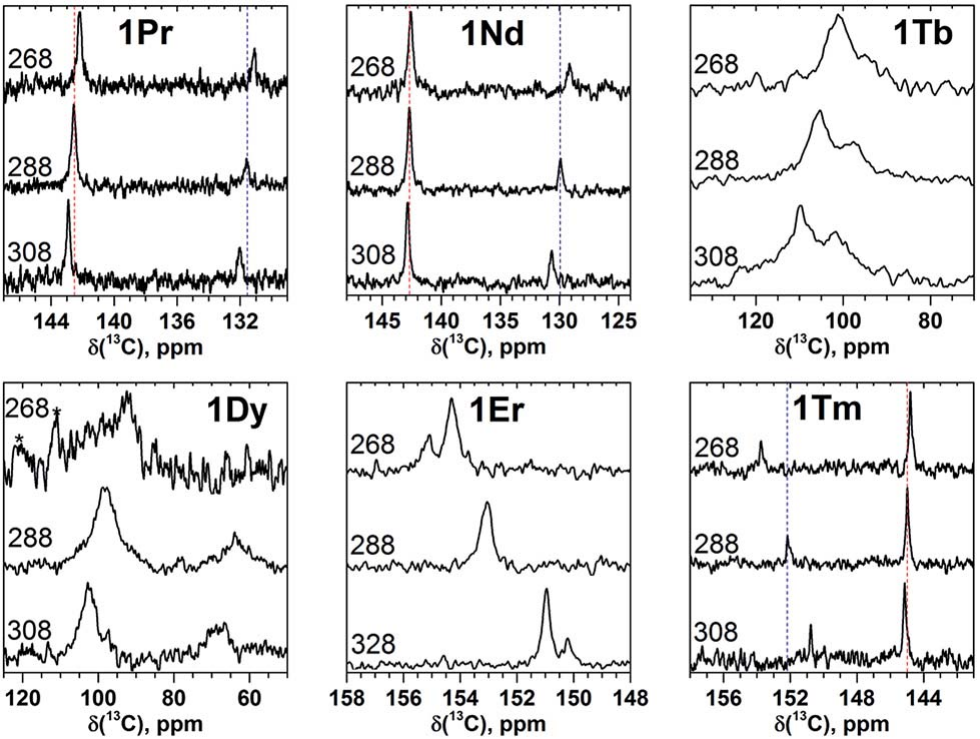
Variable-temperature ^13^C NMR spectra of selected **1M** compounds measured in CS_2_ in the 268–308 K range. Vertical dash lines denote chemical shifts at 288 K and are shown to guide an eye. At 288 and 308 K two carbon signals in **1Er** are not resolved, and therefore the measurements were performed in *o*-DCB in a broader temperature range (268–328 K). Low solubility of **1Dy** and **1Tb** at 268 K resulted in poor signal-to-noise ratio (the noise features in the spectrum of **1Dy** are marked by asterisks).

**Fig. 5:**
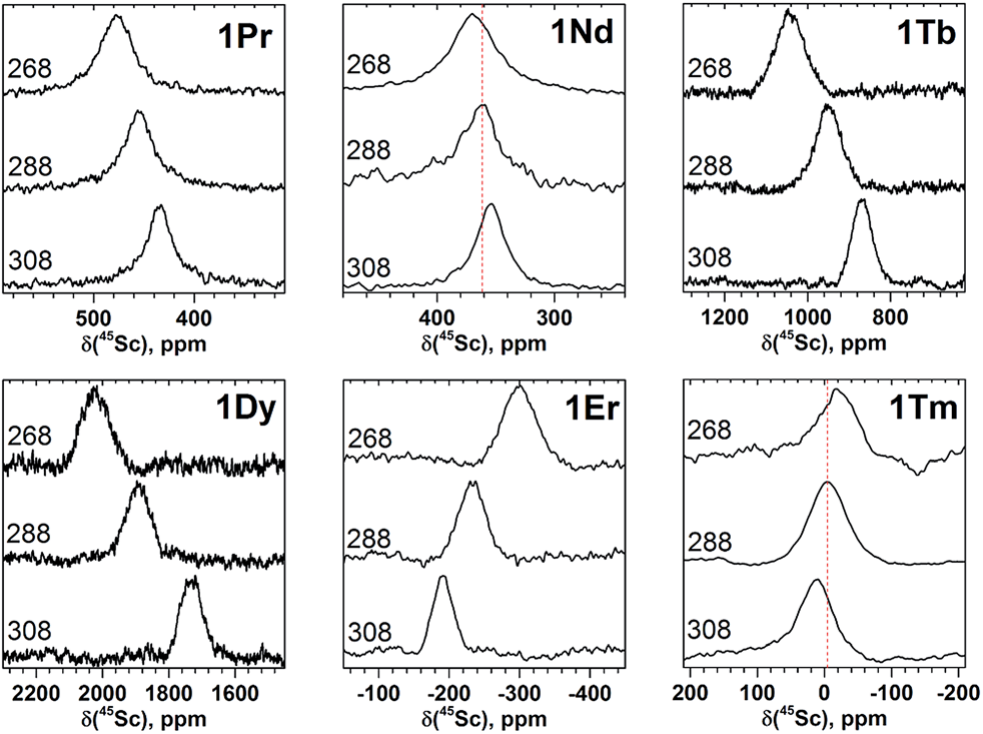
Variable-temperature ^45^Sc NMR spectra of selected **1M** compounds measured in CS_2_ in the 268–308 K range. To guide an eye, vertical lines in the spectra or **1Nd** and **1Tm** indicate signal positions at 288 K.

Temperature dependence of paramagnetic NMR shifts can be used as an additional parameter to distinguish the contact and pseudocontact contributions as well as to clarify the extent of the magnetic anisotropy. Fig. 4 and 5 show variations of ^13^C and ^45^Sc NMR spectra in the temperature range 268–308 K. To quantify the temperature variation, we will use the Δ308268(*δ*) parameter, which is the difference of the chemical shifts measured at 308 and 268 K.

Temperature variation of the peak positions is in line with the single-temperature *δ* ^para^ values. Namely, the compounds with strong paramagnetic shifts exhibit enhanced variation of the peak position with the temperature and *vice versa*. Likewise, the sign of the Δ308268(*δ*) values is opposite to the sign of the *δ* ^para^ shifts. The only exclusion is the PHHJ signal of **1Tm**, for which both *δ* ^para^ and Δ308268(*δ*) parameters are positive. Since this signal has the smallest *δ* ^para^ value (0.79 ppm), we cannot exclude that uncertainties in the estimation of the *δ* ^dia^ value may play a role here. More likely, however, is that the pseudocontact and contact contributions have opposite signs and compensate each other in this molecule.

#### Line width and relaxation times

Variation of the line width, which is especially well pronounced in the ^13^C NMR spectra, points to the substantial change of relaxation times across the lanthanide row. A transverse relaxation time *T*_2_ can be estimated using the relation *T*_2_ = (*π*Δ*ν*_1/2_)^–1^, where Δ*ν*_1/2_ is the peak width at its half maximum. The Δ*ν*_1/2_ values determined for PHHJ signals at 288 K are listed in Table 1. For **1Tm** we have also measured *T*_2_ and *T*_1_ times directly using Carr–Purcell–Meiboom–Gill pulse sequence and inversion recovery method, which gave 50 ms and 66 ms at 288 K, respectively (see ESI Fig. S7 and S8† for other temperatures). As might be anticipated, *T*_1_ is longer than *T*_2_, and the *T*_2_* value of 19 ms determined by the line width method underestimates the real *T*_2_ time but gives correct order of the value. Direct measurements of shorter relaxation times is beyond the possibilities of our spectrometer. In diamagnetic **1M** compounds, the nuclei spin relaxation times are much longer.^42^ For instance, *T*_2_ and *T*_1_ times of the PHHJ carbons in **1Y** are 17 and 23 s, respectively. Note that *T*_2_ determined from the CPMG pulse sequence for diamagnetic **1Y** is significantly longer than might be estimated from the line width (*T*_2_* = 0.2 s), which shows that the line width for such narrow signals is limited by the instrument.

Comparison of the Δ*ν*_1/2_ values for ^13^C peaks in **1M** series (Fig. 1 and Table 1) reveals dramatic influence of the lanthanide on the nuclei spin relaxation. For instance, ^13^C nuclei spins in **1Dy** (*T*_2_ ≈ 0.3 ms) relax *ca.* 5 × 10^4^ times faster than in diamagnetic **1Y** (*T*_2_ = 17 s). The plot of Δ*ν*_1/2_ *versus δ* ^para^ for the PHHJ carbons along the **1M** series can be well fit by a parabola (see ESI Fig. S9†). This fact points to the prevailing contribution of the pseudocontact shift in the total paramagnetic shift since the relaxation rate in paramagnetic molecules has *R* ^–6^ dependence on the distance between the nuclei and paramagnetic center, whereas the pseudocontact shift scales as *R* ^–3^ (eqn (1)).

The broadening of ^45^Sc signals along the **1M** series is not so pronounced as for carbon signals because the ^45^Sc quadrupole moment broadens the peaks even in diamagnetic molecules. The ^45^Sc signals in **1Dy**, **1Tb**, **1Ho**, and **1Tm** are roughly two times broader than in the **1Y** (Fig. 3), which can be ascribed to a paramagnetic effect. At the same time, in **1Ce**, **1Pr**, **1Nd**, and **1Er** the line width is comparable to that of diamagnetic **1Y**, which means that the spin relaxation rate in these molecules is still determined by a quadrupole effect.

The line width exhibits substantial decrease at lower temperatures (Fig. 4 and 5). For instance, ^45^Sc signals measured at 268 K are roughly twice broader than at 308 K (Fig. 5). In some previous works, temperature dependence of the ^45^Sc line width was used to estimate rotation barrier of the endohedral clusters.^19,43^ If the line width is directly proportional to the rotational correlation time, the plot ln(Δ*ν*_1/2_)-*versus-T* ^–1^ gives a straight line with the slope proportional to the activation energy. In this work, none of the **1M** molecules gave straight line in the ln(Δ*ν*_1/2_)-*versus-T* ^–1^ coordinates, preventing thus further analysis of the data. The broadening at low temperature should result from the interplay of the slowing of the cluster dynamics and the electron spin contribution, which can hardly be separated at this moment.

### Separation of contact and pseudocontact shifts

The localized “buried” nature of the 4f electrons in lanthanides implies that the contact contribution to the paramagnetic shift should be rather small. Typically, the values become negligible when the nucleus of interest is separated by more than four bonds from the lanthanide ion. However, in the **1M** molecules both Sc and carbon atoms are located rather close to the lanthanide, and hence the contact term cannot be ignored. With systematic NMR data along the whole lanthanide series in hand, we can address this problem. Separation of the contact and pseudocontact shifts is a long standing problem in paramagnetic NMR, and several schemes were proposed to solve it.^11c,13,44^ Unfortunately, all methods allowing analytical deviation of linearizable equations are based on Bleaney's assumption^45^ that LF splitting is less than the thermal energy and hence expansion of the magnetic susceptibility in reciprocal temperature series is limited to *T* ^–2^. This assumption is definitely not fulfilled for **1M** molecules, and hence the results of these calculations may give only qualitative estimation at best (see also ref. 41a for a detailed discussion of the limitations of Bleaney's theory). Another assumption usually applied is that the system is either purely axial (LF operator described in phenomenological *B*_*q*_^*k*^ parameters is limited to the *B*_0_^2^ term), or its rhombic anisotropy can be described solely by a *B*_2_^2^ parameter. The sum of contact and pseudocontact shifts can be then described as:

2 *δ*para*ij* = *F*_*i*_*S*_*z*__*j*_ + *C*_*j*_(*B*_0_^2^*G*_*i*_ + 6*B*_2_^2^*H*_*i*_)

The first term is the contact shift, where *F*_*i*_ is proportional to a Fermi coupling constant, and *S*_*z*__*j*_ is the expectation value of the spin projection operator *S*_*z*_ for a given lanthanide. The second term is the pseudocontact shift, *C*_*j*_ is a numerical factor specific for each lanthanide and tabulated by Bleaney *et al.*,^45*b*^ whereas *G*_*i*_ and *H*_*i*_ are structural factors (compare to eqn (1a)). If rhombic anisotropy is negligible, the second term reduces to *δ*pc*ij* = *C*_*j*_ *B*_0_^2^ *G*_*i*_ (compare to eqn (1b)).

#### Reilley's method

Following the method proposed by Reilley *et al.*,^13^eqn (2) can be written in the form:3
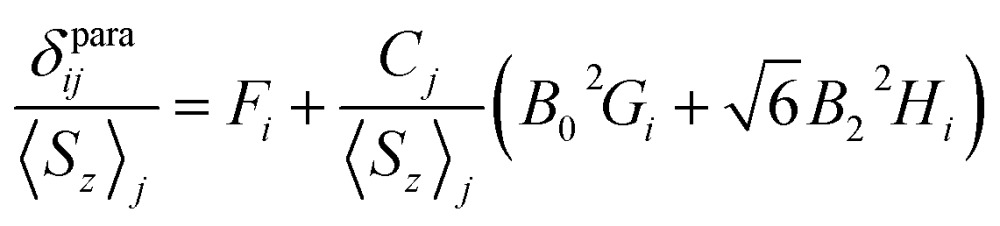



The plot *δ*para*ij*/*S*_*z*__*j*_ *versus C*_*j*_/*S*_*z*__*j*_ along the lanthanide series is expected to give a straight line, if all molecules are isostructural, and the *F*_*i*_ value and LF parameters are not changing along the series. Contact and pseudocontact shifts can be then computed from the intercept and the slope.

**Fig. 6:**
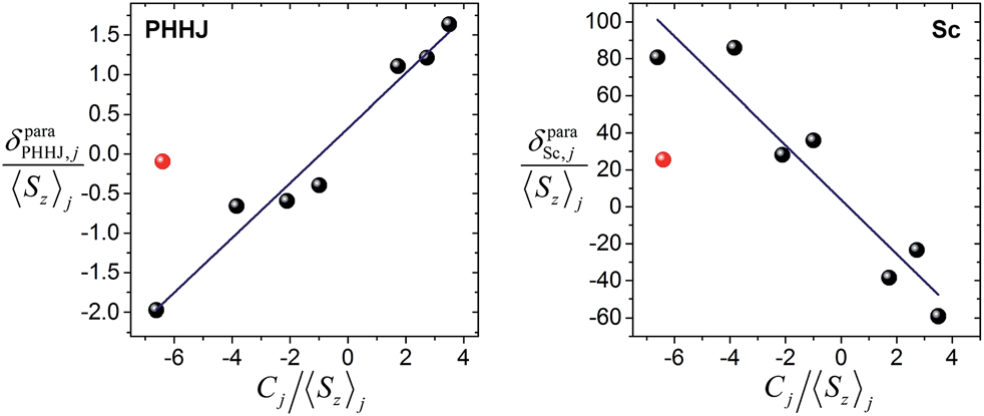
Correlations between *δ*para*ij*/*S*_*z*__*j*_ and *C*_*j*_/*S*_*z*__*j*_ for ^13^C-PHHJ (left) and ^45^Sc (right) signals used to distinguish contact and pseudocontact contributions in **1M** molecules by Reilley's approach. **1Tm** values are shown as red dots; they were not included in the linear fit. The quality of linear fit is *R* ^2^ = 0.97 for ^13^C-PHHJ and *R* ^2^ = 0.90 for ^45^Sc.

Fig. 6 shows *δ*para*ij*/*S*_*z*__*j*_ *versus C*_*j*_/*S*_*z*__*j*_ plots for two NMR signals measured for **1M** molecules. ^13^C PHHJ and ^45^Sc shifts can be roughly linearized if **1Tm** values are excluded from the set; the *δ* ^con^ and *δ* ^pc^ shifts computed from the linear fits are listed in Tables 2 and 3. The ^13^C THJ shifts cannot be fitted by a straight line in these coordinates (see ESI Fig. S10†).

**Table 2:** Contact and pseudocontact ^13^C chemical shifts in **1M** molecules

**1M**	*S*_*z*__*j*_ ^*a*^	*C*_*j*_ ^*a*^	PHHJ	DFT^*b*^	*δ* ^pc^	DFT^*c*^	PCM^*d*^	Δ308268^*e*^	THJ	*δ* ^pc^	PCM^*d*^	Δ308268^*e*^
			*δ* ^con^						*δ* ^con^			
			Reilley		Reilley				DFT^*b*^	DFT^*c*^		
Ce	0.98	–6.48	0.3	0.4	–2.2	–2.3	–1.2	0.2	0.2	–2.4	–3.4	0.5
Pr	2.97	–11.41	1.0	1.2	–4.0	–3.1	–2.7	0.4	0.8	–6.9	–7.7	1.2
Nd	4.49	–4.46	1.4	1.8	–1.5	–3.6	–2.1	0.5	1.1	–8.9	–6.0	1.4
Tb	–31.82	–86.84	–10.3	–12.7	–30.1	–25.9	–17.6	3.1	–8.1	–31.2	–61.2	10.9
Dy	–28.54	–100	–9.2	–11.4	–34.6	–35.2	–24.2	4.3	–7.3	–68.3	–73.0	12.9
Ho	–22.63	–39.25	–7.3	–9.0	–13.6	–16.0	–12.5	3.4	–5.8	–57.2	–47.3	12.0
Er	–15.37	32.4	–5.0	–6.1	11.2	15.3	5.8	–1.3	–3.9	20.1	21.0	–4.6
Tm	–8.21	52.53	–2.6	–3.3	18.2	4.1	5.9	–1.1	–2.1	17.1	17.8	–2.8

^*a*^ *S*_*z*__*j*_ and *C*_*j*_ values are adopted from ref. 44a.

^*b*^PBE/SARC-TZVP calculations.

^*c*^The values are obtained as *δ* ^pc^(DFT) = *δ* ^para^(exp) – *δ* ^con^(DFT).

^*d*^“PCM” stands for point charge model.

^*e*^Δ308268 values are computed using PCM approach and include only *δ* ^pc^ contribution.

**Table 3:** Contact and pseudocontact ^45^Sc chemical shifts in **1M** molecules

**1M**	δ^con^	2-nuc^*a*^	δ^pc^	2-nuc^*b*^	PCM^*c*^	Δ308268^*d*^
	Reilley		Reilley			
Ce	4	0	95	79	87	–14
Pr	11	–1	168	257	197	–31
Nd	17	–2	66	163	150	–36
Tb	–122	14	1278	735	1430	–255
Dy	–110	13	1472	1679	1799	–319
Ho	–87	10	578	862	1060	–290
Er	–59	7	–477	–440	–458	100
Tm	–32	4	–773	–213	–394	62

^*a*^Obtained using the fit of eqn (4a) together with ^13^C-PHHJ shifts, and then subtracting *δ* ^con^(^13^C) values estimated by Reilley's approach; **1Pr** value was not included in the fit; inclusion of **1Pr** shift worsened the fit from *R* ^2^ = 0.96 to *R* ^2^ = 0.87, but gave the values closer to those obtained by Reilley's approach.

^*b*^The values are obtained as *δ* ^pc^(2-nuc) = *δ* ^para^(exp) – *δ* ^con^(2-nuc).

^*c*^“PCM” stands for point charge model.

^*d*^Δ308268 values are computed using PCM approach and include only *δ* ^pc^ contribution.

The absolute values of *δ* ^con^ shifts for the PHHJ carbons are 2–4 times smaller than those of *δ* ^pc^. Furthermore, the *δ* ^con^ and *δ* ^pc^ shifts of **1Ce**, **1Pr**, **1Nd**, **1Er**, and **1Tm**, have opposite signs. For **1Nd** this analysis predicts mutual compensation of *δ* ^con^ and *δ* ^pc^ shifts, but uncertainties of 1–2 ppm make the analysis of the small values rather ambiguous.

The ratio between *δ* ^con^ and *δ* ^pc^ for the ^45^Sc chemical shifts is smaller than for the ^13^C counterparts, and the contact term contribution drops to less than 10% for the majority of lanthanides; the largest relative *δ* ^con^ values of *ca.* 20% are found in **1Nd** and **1Ho**. Thus, although uncertainty of the fit is rather high, the dominance of the pseudocontact term is beyond any doubt.

Deviations from the straight line in eqn (3) are usually considered to be an indication of the loss of the isostructurality along the series. This can hardly be the case for the **1M** series since molecular structures determined by single-crystal X-ray diffraction for some of them are very similar (subject to vary with lanthanide contraction, see below).^19,26–28^ More likely, other assumptions used in the model are not valid. In particular, Reilley's approach implies that the LF parameters are not changing along the lanthanide row, which is most probably not correct for the **1M** molecules.^7*b*^

#### Parameter-free models

The requirement of the constant LF parameters is one of the obvious weak points of Reilley's approach, which can be circumvented by combining the data on two or more nuclei and excluding *B*_0_^2^ and *B*_2_^2^ terms in eqn (3). For an uniaxial system (*B*_2_^2^ = 0), the 2-nuclei parameter-free model is obtained by plotting *δ*para*ij*/*S*_*z*__*j*_ *versus δ*para*kj*/*S*_*z*__*j*_, which should give a straight line for the isostructural series (ref. 46):4a
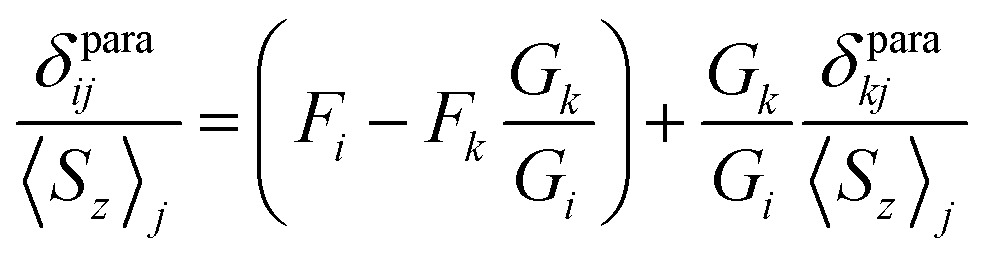



The slope of the line is a ratio of the structural factors *G*_*i*_/*G*_*k*_, and the intercept is a difference of *F*_*i*_ and *F*_*k*_ *G*_*i*_/*G*_*k*_. For the molecules with a rhombic anisotropy (*B*_2_^2^ ≠ 0), the parameter-free model can be obtained in a similar fashion by simultaneous analysis of chemical shifts of three different nuclei. In the latter case linearization gives the equation of a plane (ref. 47):4b
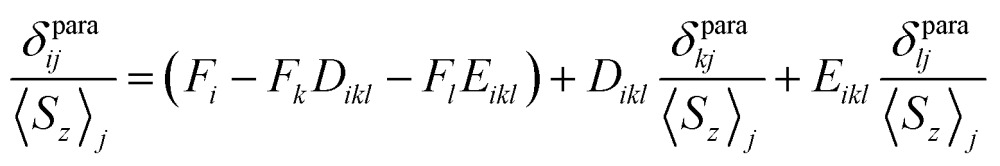
here coefficients *D*_*ikl*_ and *E*_*ikl*_ are functions of the structural parameters *G*_*i*_ and *H*_*i*_ from eqn (3), see ref. 47a for full details.

**Fig. 7:**
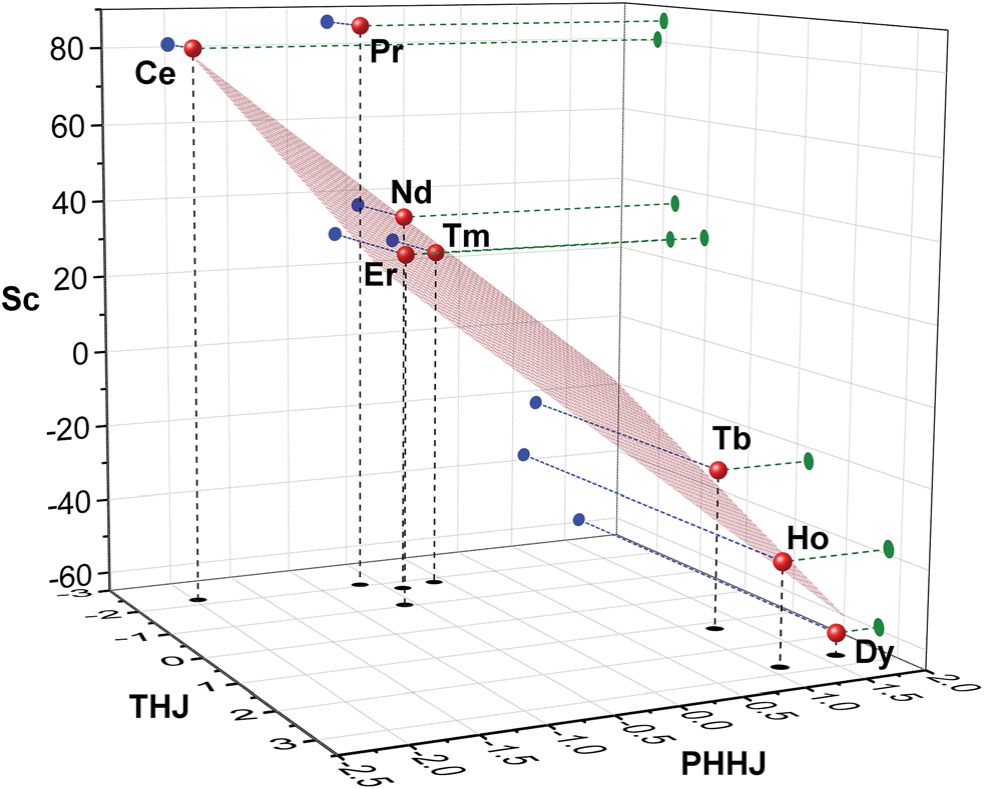
Three-nuclei 3D plot in *δ*para*ij*/*S*_*z*__*j*_ coordinates for ^45^Sc, ^13^C-THJ and ^13^C-PHHJ chemical shifts in **1M** series (*T* = 288 K). Red spheres are 3D data; blue, green, and black circles are projections on coordinate planes. Red grid shows a fitted plane (1Pr was not included in the fit).

Fig. 7 visualizes analysis of all chemical shifts for **1M** molecules within parameter free models. The 3D plot corresponds to the 3-nuclei method, whereas projections on the coordinate planes allow evaluation of the 2-nuclei method for three different combination of nuclei. In the 3-nuclei model, all data points except for **1Pr** are close to the plane obtained by the least square fitting of eqn (4b). Analysis of the 2D plots in Fig. 7 shows that a good linear correlation exists only for the PHHJ-*vs.*-Sc plot (**1Pr** value is deviating again). Substituting the *δ* ^con^ values obtained for the PHHJ carbons using eqn (3) into eqn (4a) for the PHHJ-*vs.*-Sc correlation allows estimation of the ^45^Sc *δ* ^con^ shifts. The values obtained by this way are much smaller than those obtained using Reilley's approach. It should be also noted that the results of the *δ* ^con^ calculations for Sc using the 2-nuclei method are rather erratic and change significantly with small variation of the input parameters. At the same time, both methods agree in that the ^45^Sc *δ* ^con^ shifts are considerably smaller than the pseudocontact contributions.

The fact that the data points for THJ carbons fit well into the 3-nuculei model but do not give linear correlations in the 2-nuclei model might indicate that the rhombic anisotropy contribution is considerable here, but the limited amounts of points does not allow more solid conclusions.

#### DFT calculations of contact shifts

Independent computations of contact shifts are possible using quantum chemical approaches. DFT computations of lanthanide-containing molecules for systems other than 4f^7^ cannot be reliable (unless 4f-in-core effective core potentials are used, but this approach cannot be used if spin properties related to the 4f electrons are of interest). However, the *F*_*i*_ value usually remains the same for the whole lanthanide row, and hence computations can be performed for Gd analogues. Recently, such calculations provided accurate estimation of the contact shifts in a series of Tb complexes.^48^

**Fig. 8:**
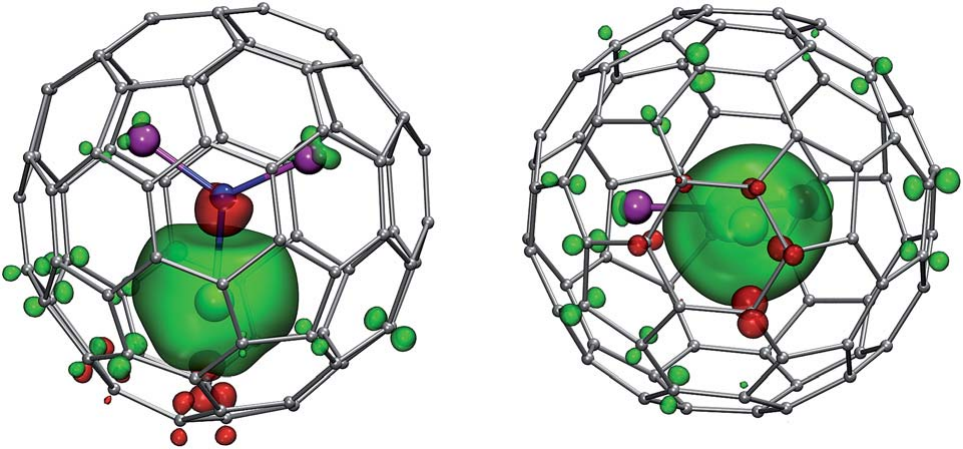
Spin-density distribution in GdSc_2_N@C_80_ (two orientations of the molecule are shown). To visualize small spin polarization effects, the isosurfaces are plotted at relatively small spin density values of ±0.0006 a.u. (green (+) and red (–)).

Fig. 8 shows the spin density distribution in **1Gd** computed using the scalar-relativistic DKH2 approximation, PBE functional, and SARC-TZVP basis set. Negative spin polarization of the central nitrogen atoms and several carbon atoms close to the lanthanide can be well seen (red-colored lobes). At the same time, the sign of the spin density on the Sc atoms and carbon atoms further away from the Gd ion is the same as for the Gd itself.

Hyperfine coupling constants for carbon atoms in GdSc_2_N@C_80_ span the range from –0.041 MHz to 0.096 MHz, and the averaging over the whole carbon cage yields the values of 0.015 and 0.010 MHz for the PHHJ and THJ carbons, respectively. The pseudocontact shifts computed for ^13^C atoms using these constants are listed in Table 2. Remarkably, the DFT-computed values for the PHHJ carbons are quite close to those obtained by Reilley's method. If we tentatively suggest that the agreement between the theory and experiment is equally well for the THJ carbons, their contact shifts should be 35% smaller than those of PHHJs. Furthermore, relative contributions of the contact term in total paramagnetic shifts of the THJ carbons are much smaller than for the PHHJ carbons.

For the ^45^Sc hyperfine coupling constant, the PBE-DKH approximation with tailored SARC-TZVP basis set gives the average value of –0.27 MHz. An increasing the basis set to def2-TZVP or inclusion of the spin–orbit coupling corrections give the values of –0.26 MHz and –0.28 MHz, respectively, whereas switching to ZORA scalar relativistic method reduces the ^45^Sc hyperfine coupling constant to –0.21 MHz. If hybrid PBE0 functional is used instead of PBE with SARC-TZVP basis, predicted DKH and ZORA values are –0.35 and –0.49 MHz, respectively. The use of PBE-DKH/TZVP constants for estimation of the ^45^Sc contact shifts in **1M** molecules yields unrealistically high values (625 ppm for **1Tb**, 560 ppm for **1Dy**, *etc.*). It should be noted that computation of the ^45^Sc hyperfine coupling constants is less straightforward than for ^13^C nuclei because of the subtle balance between the valence- and core–shell spin polarizations, which often have opposite signs in transition metals.^49^ Due to this reason, prediction of the ^45^Sc hyperfine coupling constants meets serious difficulties even for the Sc-based EMF radicals such as Sc_3_N@C_80_^–^ (ref. 50), where coupling constants are large and dominated by SOMO contribution. Reliable prediction of the core–shell spin polarization becomes even more problematic for paramagnetic molecules, in which the spin density on the Sc atoms is largely induced by spin polarization (such as in **1M**). We have to admit that DFT prediction of small ^45^Sc coupling constants in **1M** can hardly be reliable at this moment and refrain from further discussion of DFT-computed *δ* ^con^(^45^Sc) values.

#### Temperature dependence of chemical shifts

If Bleaney's conditions are satisfied, the temperature dependence of chemical shifts can be also used to distinguish contact and pseudocontact terms: whereas the contact term scales as *T* ^–1^, the pseudocontact term has *T* ^–2^ dependence. If one of the terms dominates, plot *δ* ^para^ *versus T* ^–1^ or *T* ^–2^ should give a straight line, whose intercept is close to *δ* ^dia^. In due turn, a large deviation of the intercept from the reference *δ* ^dia^ value signals that the corresponding contribution is small. This simple yet efficient approach was employed before to prove the dominance of pseudocontact shift in Ce-based EMFs. However, this approach can give reliable results only if the pseudocontact term has real *T* ^–2^ dependence, and if one the terms dominates. If LF splitting is larger than the thermal energy, contribution of *T* ^–3^ and higher terms cannot be ignored,^51^ and results of the linear fitting become inconclusive. In the **1M** series, only for **1Ce** is the intercept of the *δ* ^para^(*T* ^–2^) plot close to *δ* ^dia^ for all three nuclei (see ESI Table S1†). For all other lanthanides, the intercepts of *δ* ^para^(*T* ^–1^) and *δ* ^para^(*T* ^–2^) linear fits give rather irregular set of data, sometimes with large deviations from *δ* ^dia^ for both *T* ^–1^ and *T* ^–2^ plots (ESI Table S1†). Thus, large magnetic anisotropy of lanthanide ions in **1M** molecules prevents the use of temperature dependence for distinguishing contact and pseudocontact contributions to paramagnetic chemical shifts. Good results obtained for the **1Ce** in *δ* ^para^(*T* ^–2^) fits are in part due to the small *S*_*z*__Ce_ value (Table 2), which makes the contact term negligible.

### Geometry parameters of **1M** molecules

**Fig. 9:**
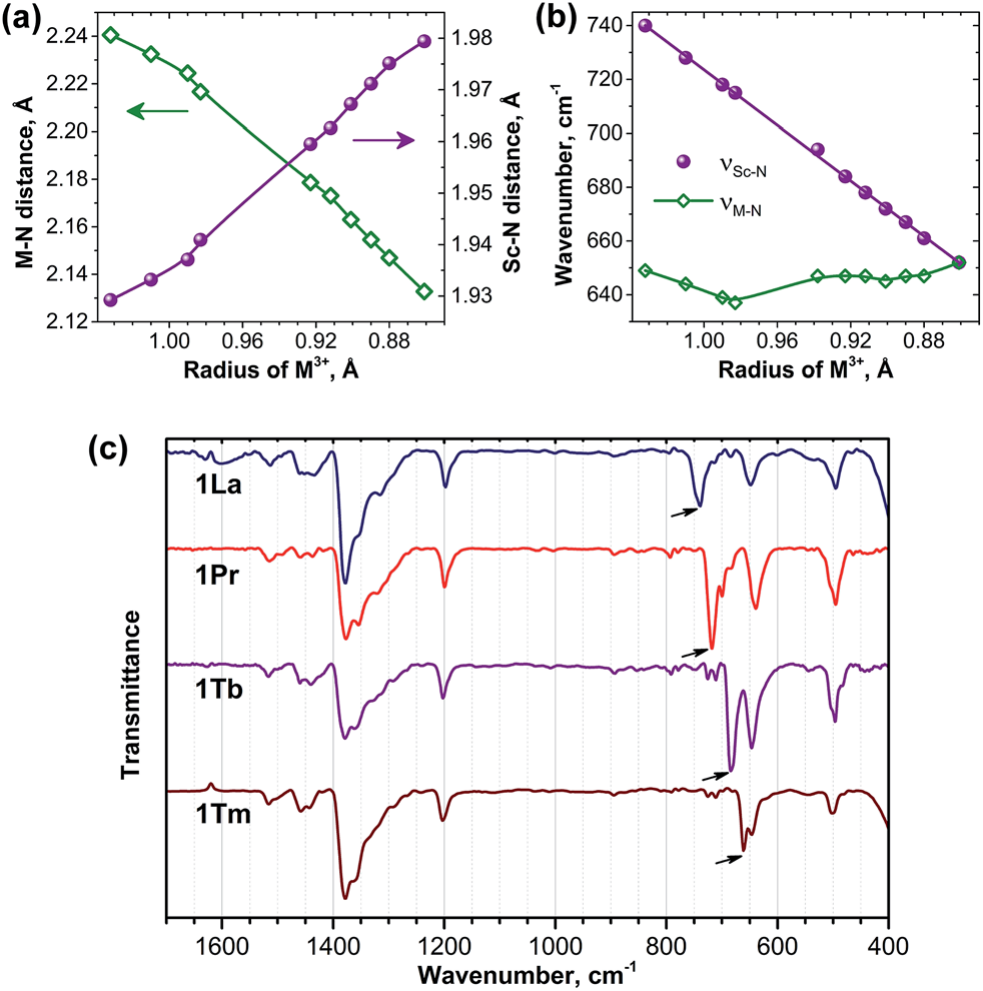
(a) Correlation between DFT-computed M–N and Sc–N bond lengths in **1M** molecules and ionic radii of lanthanides; (b) correlation between the metal–nitrogen stretching mode frequencies and ionic radii of lanthanides (green – M–N bonds, purple – Sc–N bonds); (c) FTIR spectra of selected **1M** molecules: **1La**, **1Pr**, **1Tb**, and **1Tm**; arrows mark the *ν*_Sc–N_ modes.

For the further analysis of the magnetic state and the LF splitting of lanthanide ions in the **1M** compounds it is necessary to take into account a variation of the molecular geometry parameters along the series. Namely, a significant decrease of the ionic radii of lanthanides from La to Lu cannot be ignored, especially when magnetic anisotropy is large. Therefore, to obtain a consistent set of the molecular geometry parameters along the whole 4f row for the subsequent LF calculations (see next section), we performed DFT optimization of all structures using the B3LYP functional and 4f-in-core basis set for lanthanides. The M–N and averaged Sc–N bond lengths are listed in ESI Table S2† and are plotted *versus* Shannon ionic radii of M^3+^ ions in Fig. 9. As expected, the decrease of the ionic radii from La to Lu results in the gradual decrease of the M–N distance (from 2.241 Å in **1La** to 2.133 Å in **1Lu**). At the same time, since the carbon cage remains the same, the increase of the lanthanide size also leads to the simultaneous decrease of the Sc–N bond lengths (compare 1.929 Å in **1La** to 1.979 Å in **1Lu**) so that the cluster size remains more or less the same along the whole series. Single-crystal X-ray structures were reported earlier for **1La**,^26^ **1Ce**,^19^ **1Gd**,^27^ **1Tb**,^27^ and **1Er**,^28^ and experimental bond length values also confirm this trend. Namely, in the **1La**–**1Ce**–**1Gd**–**1Tb**–**1Er** the lanthanide–nitrogen bond is decreasing as 2.196(4)–2.184(2)–2.149(10)–2.126(11)–2.089(9) Å. The averaged Sc–N distance is varying as 1.932(7)–1.938(2)–1.918(9)–1.949(8)–1.968(6) Å, respectively, *i.e.* there is a gradual decrease from **1La**/**1Ce** to **1Tb** to **1Er** (note that **1Gd** deviates from the trend). A comparison to the computed data shows that DFT overestimates the M–N distances by *ca.* 0.04–0.06 Å, whereas the error for the Sc–N distances is smaller than 0.01 Å. Overall, DFT-optimized bond lengths give reasonable estimation of the experimental values.

Significant variation of the bond lengths in nitride clusters can be also inferred from the analysis of the FTIR absorption spectra. In the frequency range of 500–800 cm^–1^, all nitride clusterfullerenes have a characteristic antisymmetric metal–nitrogen stretching vibration with medium to strong absorption intensity, whose frequency correlates with the metal–nitrogen bond length and therefore can be used for the analysis of the structural correlations.^29c,52^ This mode corresponds to the in-plane motion of the nitrogen atom and is two-fold degenerate for the M_3_N cluster with the frequency of (3*k*_M–N_)^0.5^ *m*_N_^–0.5^, where *k*_M–N_ is the force constant of the metal–nitrogen bond, and *m*_N_ is the mass of the nitrogen atom. In the mixed-metal cluster MSc_2_N, the degeneracy is lifted and the frequencies of two resulting vibrations are:

5a *ν*_Sc–N_ = (3*k*_Sc–N_)^0.5^*m*_N_^–0.5^

5b *ν*_M–N_ = (2*k*_M–N_ + *k*_Sc–N_)^0.5^*m*_N_^–0.5^

Fig. 9c compares the IR spectra for **1La**, **1Pr**, **1Tb**, and **1Tm** (IR spectra of other **1M** compounds were reported by our group earlier^20b,24,29c^). In all spectra both the *ν*_M–N_ and *ν*_Sc–N_ components can be clearly identified in the 600–800 cm^–1^ range. Comparison of the *ν*_M–N_ values along the **1M** series is not straightforward, because the frequency *versus* bond length correlation does not hold exactly for different metals and also because of the admixture of the *k*_Sc–N_ force constant in eqn (5b). In fact, the *ν*_M–N_ frequency remains in the range of 635–650 cm^–1^ in the whole **1M** series (Fig. 9b). The *ν*_Sc–N_ frequencies are better suited for the structural correlations: Fig. 9b shows that the *ν*_Sc–N_ frequencies vary from 652 cm^–1^ in **1Lu** to 740 cm^–1^ in **1La** and exhibit perfect linear correlation with the lanthanide ionic radii. Very good correlation also exists between the DFT-predicted Sc–N bond lengths and experimental *ν*_Sc–N_ frequencies (not shown), thus confirming reliability of the DFT-computed structural parameters.

### Ligand field in **1M** molecules

**Fig. 10:**
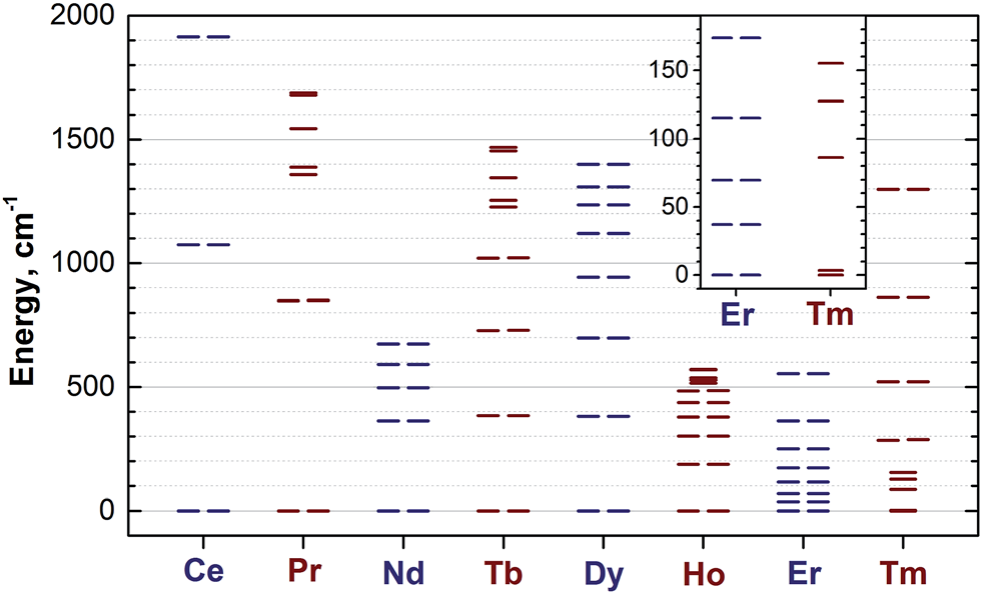
Ligand-field splitting of the *m*_J_ levels in the **1M** compounds computed using point-charge model. The inset shows low-energy levels in **1Er** and **1Tm** in the range of 0–190 cm^–1^. Each degenerate ±*m*_J_ level of Kramers ions (Ce, Nd, Dy, Er) and quasi-degenerate (within 3 cm^–1^) levels of non-Kramers ions are shown as double lines. To guide an eye, Kramers and non-Kramers ions are denoted in dark blue and wine, respectively.

Reliable determination and verification of the cluster bond lengths discussed in the previous section is crucial for the analysis of the LF in nitride clusterfullerenes. Recently we showed that the point charge model employing scaled Bader charges can reasonably reproduce the *ab initio* (CASSCF) computed ligand field splitting in DySc_2_N@C_80_,^7a,20a^ which enables the use of this simple approach for other **1M** compounds. To calculate the LF splitting in this work we used B3LYP-optimized coordinates discussed above and Bader charges for nitrogen, scandium, and four closest carbon atoms (denoted C1–C4 in Fig. 1; since the cage carbon atoms have very small contribution to the LF,^20*a*^ it is sufficient to consider only few nearest atoms, their QTAIM charges are listed in the ESI Table S2†). The charges were scaled by a factor of 0.754 to match the *ab initio* LF splitting in **1Dy** from ref. 7a (see ESI Table S3†). Fig. 10 shows an overview of the *m*_J_ levels in all studied **1M** molecules (the values and assignment are listed in the ESI Table S4†).

The central nitrogen atom gives the main contribution to the LF in nitride clusterfullerenes because of its large negative charge (Bader charge –1.7) and close distance to the lanthanide ion (see ESI Table S3† for the LF point charge calculations using different parts of the MSc_2_N@C_80_ molecule). Therefore, correct description of the molecular geometry is crucial since even small variations of the metal–nitrogen distance lead to considerable changes in the ligand field.^20*a*^ Two Sc ions produce the second largest contribution to the LF after the nitride ion. Their positive charges (Bader charge of *ca.* +1.7) reduce the effect of the nitride ion (*e.g.*, in **1Dy** the LF splitting is reduced by *ca.* 35% when Sc contribution is “turned on”) and introduce a deviation from the uniaxial symmetry. The charges of carbon atoms are much smaller than those of N and Sc (below –0.1), and hence only few carbons close to the lanthanide ion have non-negligible contribution to the LF (in comparison to the M + N + 2Sc system, addition of four carbons increases the splitting energies by *ca.* 12%, see ESI Table S3† for **1Dy** and ref. 20a for a more detailed analysis of **1Ho**). Thus, the LF experienced by lanthanide ions in **1M** molecules has roughly a uniaxial character induced by a large negative point charge of the nitride ion, which results in rather simple LF splitting pattern despite the low molecular symmetry. A similar situation was found for a series of other Dy-based SMMs with low molecular symmetry.^53^

#### Lanthanide ions with oblate 4f-density

**Fig. 11:**
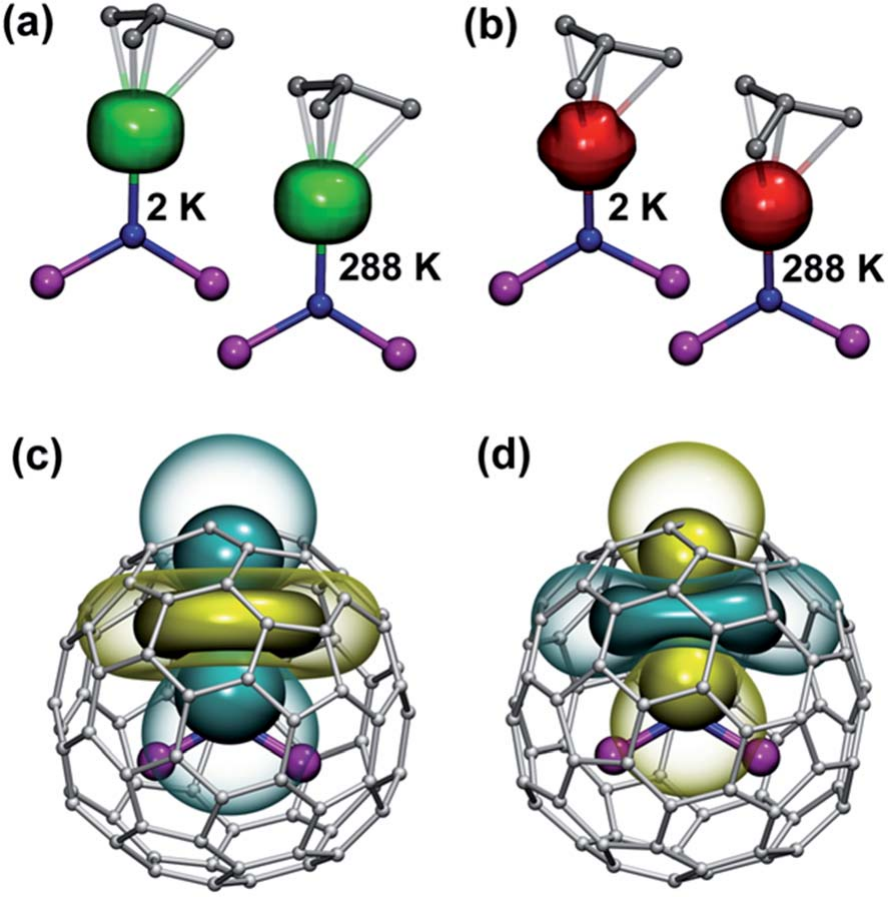
(a and b) isosurfaces of 4f electron density in **1Dy** (a, green) and **1Er** (b, red) computed at 2 and 288 K. Only the nitride cluster and four carbon atoms nearest to the lanthanide ion are shown. Whereas the shape of the lobe remains almost the same in **1Dy**, in **1Er** the increase of the temperature from 2 to 288 K changes the shape of the 4f density distribution to a more spherical one. (c and d) Pseudocontact shift isosurfaces in **1Dy** (c) and **1Er** (d) at 288 K computed using the point-charge model (cyan – positive, yellow – negative). Solid/transparent surfaces correspond, respectively, to ±6000/±1500 ppm isovalues in **1Dy** and ±2000/±500 ppm isovalues in **1Er**.

The ground magnetic state of the ions with oblate shape of the 4f electron density (Ce, Pr, Nd, Tb, Dy, Ho; see ref. 54 and Fig. 11a for 4f density in **1Dy**) has the largest *J*_*z*_ value (5/2 in **1Ce**, 4 in **1Pr**, 9/2 in **1Nd**, 6 in **1Tb**, 15/2 in **1Dy**, and 8 in **1Ho**), and the energy of the other *m*_J_ states is gradually increasing with the decrease of the |*m*_J_| values. The gaps between the neighboring *m*_J_ states are also getting smaller with the decrease of the |*m*_J_| value. The deviation from the uniaxial symmetry induced by the two Sc ions is well seen for non-Kramers ions (Pr, Tb, Ho). For these ions, degeneracy of the ±*m*_J_ states is not enforced, but remains rigorous in the uniaxial ligand field produced by a single nitride ion. When the contribution of the Sc ions is also taken into account, an approximate degeneracy of the ±*m*_J_ states holds only for large projections of the total momentum, whereas for the levels with |*m*_J_| = 1–2 the splitting becomes significant. A mixing of the *m*_J_ states is also substantial for small |*m*_J_| values showing that the *m*_J_ is not a “good” quantum number anymore.

The practical importance of the states with small |*m*_J_| values in ions with oblate 4f density is rather limited because the LF splitting in **1M** is very large (Fig. 10). The smallest overall LF splitting, 571 cm^–1^, is found in **1Ho**, whereas the largest LF splitting is reaching 1915 cm^–1^ in **1Ce**. The gap between the ground state and the first excited state in **1Ce** (1075 cm^–1^), **1Pr** (849 cm^–1^), **1Nd** (364 cm^–1^), **1Tb** (384 cm^–1^), and **1Dy** (382 cm^–1^) is so large that even at room temperature the ground magnetic state with the largest *J*_*z*_ projection is predicted to have predominant contribution to the magnetic properties. As a result, 4f density in such **1M** molecules exhibits only small variation with temperature. For instance, 4f densities in **1Dy** calculated at 2 K and 288 K can hardly be distinguished by eye (Fig. 11a).

The two-fold degenerate ground state with the large *J*_*z*_ projection and a significant gap to the first excited state are prerequisites for the SMM behaviour,^54*b*^ and hence the data in Fig. 10 show that slow magnetization reversal can be expected for all **1M** compounds (except for the **1Gd**, **1Er** and **1Tm**). The SMM behaviour with exceptionally long attempt time is already discovered for **1Dy**.^3,4^ Field-induced SMM behaviour is also found in non-Kramers **1Ho**,^5^ albeit on a much shorter timescale than in **1Dy**. The rigorous protection of the two-fold degeneracy of the ground magnetic state in Kramers ions makes them more attractive for single ion magnetism, and we expect **1Ce** and **1Nd** to be the next candidates for the SMM behaviour in nitride clusterfullerenes as already detected in some organometallic complexes of Ce^55^ and Nd.^56^ It should be however noted that the magnetization reversal barrier in **1Dy** is smaller than expected from its LF splitting,^4^ which indicates that the mechanism for the magnetization reversal in **1M** compounds is more complex and may include, for instance, Raman-like process.^5^

#### Lanthanide ions with prolate 4f-density

The LF splitting pattern in **1Er** and **1Tm** is completely different because of the prolate shape of the 4f electron density of these lanthanides (Fig. 11b). The ground state for these lanthanides has the lowest *J*_*z*_ projection (*m*_J_ = ±1/2 in **1Er** and *m*_J_ = 0 in **1Tm**). The energy is increasing with the increase of the |*m*_J_| values, and the density of states is higher in the low energy part of the spectrum, whereas at higher energies the energy levels are distributed sparser. As a result of such level distribution, variation of the temperature has profound effect on the magnetic anisotropy of **1Tm** and **1Er**. As can be seen in Fig. 11b, an increase of the temperature dramatically changes 4f electron density distribution in **1Er** and makes it almost isotropic. Thus, the room-temperature magnetic properties of **1Er** and **1Tm** result from the contribution of several *m*_J_ states.

**1Er** is the only compound in the whole **1M** series for which spectroscopic data on the lanthanide-based luminescence are available. At helium temperatures the luminescence spectra of **1Er** exhibit the fine structure due to the Er-based transitions in the ^4^ *I*_13/2_ → ^4^ *I*_15/2_ manifold.^9b,57^ Unfortunately, emission from several molecular/cluster sites complicates the fine structure and makes assignment less straightforward. The energy gap between the two lowest energy LF states determined in ref. 57, 28/37 cm^–1^, compares reasonably to the 37 cm^–1^ gap between the *m*_J_ = ±1/2 and *m*_J_ = ±3/2 states in our calculations, however the overall splitting determined in the optical measurements, 330 cm^–1^, is considerably smaller than the value of 554 cm^–1^ predicted by the point-charge model.

### Computed *versus* experimental pseudocontact chemical shifts

LF calculations allow direct estimation of the magnetic susceptibility tensor at different temperatures using Van Vleck formulae, and therefore enable computations of the pseudocontact chemical shifts using eqn (1a) or (1b) given the atomic coordinates are known (*e.g.* from DFT calculations) and they are not changing with temperature. Comparison between experimental and computed chemical shifts can be used then to evaluate reliability of the computed ligand field splitting levels. Rotation of the nitride cluster inside the carbon cage means that the values computed for individual carbon atoms should be averaged. Recently we found that the static model (simple averaging of the values) and the averaging of the values computed over molecular dynamics trajectory give comparable results for **1Ho**,^20*a*^ and therefore only static model is applied in this work.

In the **1M** molecules with oblate 4f-density eqn (1a) and (1b) give identical values of the pseudocontact shift because *χ*_*xx*_ and *χ*_*yy*_ components of the magnetic susceptibility tensor are virtually equal. In the **1Er** and **1Tm** molecules, the difference between *χ*_*xx*_ and *χ*_*yy*_ values is rather large, and hence eqn (1a) is to be used. The pseudocontact shifts computed for the THJ and PHHJ carbons as well as for the Sc atoms at *T* = 288 K are listed in Table 2 and 3 along with the computed Δ308268(*δ*) values. Fig. 11 plots pseudocontact shift isosurfaces in **1Dy** and **1Er** as examples of lanthanides with oblate and prolate 4f density shapes, respectively.

**Fig. 12:**
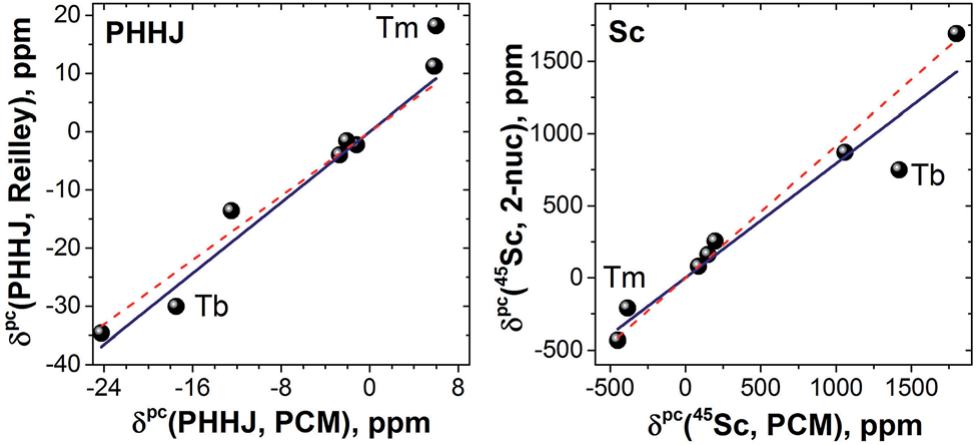
Correlation between computed and experimental chemical pseudocontact shifts: ^13^C-PHHJ (left) and ^45^Sc (right). Experimental *δ* ^pc^(^13^C) shifts are obtained by Reilley's approach, whereas ^45^Sc values are estimated using 2-nucleus method, see text for further details. Solid lines is a linear fit for a complete set of data (*R* ^2^ = 0.94 for both PHHJ and Sc), whereas red dashed lines were obtained for fitting without **1Tm** and **1Tb** values. The intercept was set to zero in linear fits.

The reliability of the point charge model in calculations of the magnetic susceptibility can be evaluated comparing experimental and computed *δ* ^pc^ shifts (Fig. 12). As experimental values we use the results of Reilley's approach for ^13^C-PHHJ signal and the values from the 2-nuclei fitting for ^45^Sc shifts. Details for the ^13^C-THJ shifts are not discussed in the text but can be found in the ESI Fig. S11.†

As can be seen in Fig. 12, reasonably good correlation exists between experimental and computed data. If **1Tm** and **1Tb** are not included in the set, the linear correlation with the intercept set to zero gives *R* ^2^ values of 0.98 and 0.99 for the PHHJ and ^45^Sc shifts, respectively. Note also that the ^45^Sc *δ* ^pc^ shifts from the 2-nuclei model are very close to the total *δ* ^para^ shifts. Thus, we can tentatively use ^45^Sc chemical shifts of paramagnetic mixed-metal nitride clusterfullerenes to evaluate the size of the magnetic anisotropy of the endohedral lanthanide ions without rather complicated and sometimes hardly possible estimation of the contact term.

It should be also noted that the slope of the linear fit in Fig. 12 is close to 0.9 for ^45^Sc but is near 1.4 for ^13^C-PHHJ. The slope for an analogous fit for the ^13^C-THJ signals (see ESI Fig. S11†) is close to 0.9. As follows from eqn (1) and our earlier studies of the Ho-based NCFs,^20*a*^ the structural factor also plays an important role in determining the *δ* ^pc^ values, and dynamic nature of the ^13^C shifts in **1M** molecules (*i.e.* averaging over the cluster rotation) makes calculation less straightforward than for the ^45^Sc shifts. For instance, in **1Ho** the term (3cos^2^ *θ*_*i*_– 1)/12π*R*_*i*_^3^ (denoted hereafter as *G*_*i*_) computed in this work at the B3LYP level is –3.30 × 10^–4^/–1.29 × 10^–3^ Å^–3^ for the PHHJ/THJ carbons, respectively. At the same time, our recent PBE computations using Y as a model for Ho gave the values of –7.37 × 10^–4^/–5.59 × 10^–4^ Å^–3^, respectively. Along the **1M** series, the *G*_*i*_ values for carbon atoms vary by 15–20%, from –3.91 × 10^–4^/–1.13 × 10^–3^ Å^–3^ in **1Ce** to –3.16 × 10^–4^/–1.31 × 10^–3^ Å^–3^ in **1Er**. Since the slope of the linear fit between experimental and calculated *δ* ^pc^ values for THJ carbons is closer to 1, we propose that B3LYP level gives correct values for the THJ carbons, whereas *G*_*i*_ factors for the PHHJs are underestimated (and therefore the slope is close to 1.4). Thus, the use of paramagnetic ^13^C chemical shifts for analysis of the magnetic anisotropy of the endohedral lanthanide ions meets certain difficulties caused by the uncertainties in the structural factor when endohedral cluster is rotating on the NMR time scale. On the contrary, the *G*_*i*_ term for ^45^Sc is almost independent on the level of theory employed to calculate it. Moreover, its variation along the whole **1M** series is below 1% because the shortening of the M–N distance in the cluster is balanced by the elongation of the Sc–N distance. Such indifference to the structural factor makes the ^45^Sc chemical shifts more suitable for the analysis of the magnetic anisotropy in mixed-metal nitride clusterfullerenes.

Computed Δ308268 values reasonably agree with experimental data for THJ carbon and Sc signals, but are rather far from the experimental values of the PHHJ carbons. The latter is likely to be due to the larger relative contribution of the contact shift for the PHHJ carbons, which is not taken into account in PCM computations of Δ308268. This is especially well seen for the **1Tm**: based on its magnetic anisotropy, negative Δ308268 value is expected for the pseudocontact shift, but positive for the contact shift. A small positive Δ308268 value observed experimentally for the **1Tm**-PHHJ carbon is therefore due to the compensation of both terms. On the contrary, the pseudocontact contribution in the **1Tm**-THJ chemical shift is much larger than the contact counterpart, and the experimental Δ308268 value is negative in good agreement with the results of computations.

**1Tm** and **1Tb** are important deviations from the general trend (Fig. 12). Especially noticeable is the **1Tb** value for the ^45^Sc shift, which is predicted to be much more positive than experimentally observed (Fig. 12). In other words, whereas the point charge model predicts that the LF splitting in **1Tb** and **1Dy** is quite similar (Fig. 10), from the ^45^Sc NMR spectra it follows that the LF splitting in **1Tb** should be considerably smaller. Interestingly, recent *ab initio* CASSCF calculations predict that the LF splitting in **1Tb** is indeed smaller than in **1Dy**,^7*b*^ which would give better agreement with experimental NMR shifts. Similarly, correct prediction of the experimental values for **1Tm** require reliable estimation of a considerable number of the LF splitting levels (Fig. 10), rather than only 1–2 excited states needed for other **1M** molecules. Inability of the point charge model to correctly treat all states may be also the reason of the noticeable deviations well seen for **1Tm** values in Fig. 12. Therefore, both **1Tb** and **1Tm** values provide convenient training set to be considered in future computational studies. At the current moment we can conclude that the point charge model gives qualitatively correct predictions of the LF splitting in lanthanide EMFs but fails to describe more subtle effects. More advanced approaches would be needed to address this problem,^58^ but this task goes beyond the scope of this work.

## Conclusions

In this work we have performed the first systematic paramagnetic NMR study of MSc_2_N@C_80_-*I*_h_ molecules with M running through all lanthanides capable of forming nitride clusterfullerenes (M = La, Ce, Pr, Nd, Tb, Dy, Ho, Er, Tm, Lu). Analysis of the whole set of data enabled separation of the contact and pseudocontact contributions to the paramagnetic shifts. We showed that the contact shift of ^13^C nuclei may be rather large, although pseudocontact term is still larger. For the ^45^Sc shifts, the pseudocontact term dominates. Since pseudocontact term is directly dependent on the magnetic anisotropy, the ^45^Sc NMR spectra are found to be especially useful for the studies of magnetic properties of lanthanide ions in clusterfullerenes.

Interpretation of the paramagnetic NMR data required modelling of the ligand filed splitting in MSc_2_N@C_80_ molecules, which was accomplished using the point charge model. Although quite simple, this approach provided semi-quantitative agreement with experimental data. Considerable deviations between experimental and computed chemical shifts are found only for TmSc_2_N@C_80_ and TbSc_2_N@C_80_. We showed that the main contribution to the ligand field is from the nitride ion, which results in an almost uniaxial ligand field. For the lanthanides with oblate shape of the 4f density (*i.e.* all lanthanides except for Er and Tm) the ground magnetic state in MSc_2_N@C_80_ has the largest *J*_*z*_ projection with rather large gap to higher energy states (with smaller *m*_J_ values). It shows that MSc_2_N@C_80_ molecules with Kramers lanthanide ions are especially promising in the field of single molecule magnetism. Yet, more refined treatment of the ligand field splitting might be needed to fully account for all experimental data.
