# Supplementary material for: Magnetic anisotropy of endohedral lanthanide ions: paramagnetic NMR study of MSc2N@C80-I h with M running through the whole 4f row
Source: Chem Sci. 2015 Jan 28;6(4):2328–41. doi: 10.1039/c5sc00154d (PMC5645780; doi:10.1039/c5sc00154d)
Supplement: Supplementary file 1 [file SC-006-C5SC00154D-s001.pdf]

Electronic Supplementary Material (ESI) for Chemical Science.
This journal is © The Royal Society of Chemistry 2015

Magnetic Anisotropy of Endohedral Lanthanide Ions: Paramagnetic NMR Study of $MSc_2N@C_{80}$-I_h with M running through the Whole 4f Row

Y. Zhang,[a] D. Krylov,[a] M. Rosenkranz,[a] S. Schiemenz,[a] A. A. Popov[a]*

Leibniz Institute for Solid State and Materials Research, 01069 Dresden, Germany

Electronic Supporting Information

Synthesis and separation of selected **1M** molecules 2

Fitting of diamagnetic ^{13}C chemical shifts in **1Lu**, **1Y**, and **1La** by quadratic functions of ionic radius 12

Temperature dependence of T_2 relaxation time for PHHJ carbons 12

Measured T_1 and T_2 relaxation times of PHHJ and THJ carbons in selected 1M molecules 13

Correlation between peak width and absolute paramagnetic shift for ^{13}C-PHHJ carbons 13

Correlations between $\delta_{ij}^{\text{para}}/\langle S_z\rangle_j$ and $C_j/\langle S_z\rangle_j$ for ^{13}C-THJ used in Reilley's approach 14

Diamagnetic chemical shifts estimated from T^{-1} and T^{-2} temperature dependences 14

Metal-nitrogen bond distances and QTAIM-computed atomic charges in DFT-optimized $MSc_2N@C_{80}$ molecules 15

Ligand field splitting levels and their degeneracies in **1M** molecules 16

Correlation between computed and experimental chemical pseudocontact shifts for ^{13}C-THJ 19

DFT-optimized Cartersian coordinates of $MSc_2N@C_{80}$ molecules 20

Synthesis of 1La

$LaSc_2N@C_{80}$ was synthesized by a modified Krätschmer-Huffman dc-arc discharging method in our group. A mixture of La_2O_3, Sc_2O_3, guanidine thiocyanate (GT) and graphite powder (molar ratio of La/Sc/GT/C = 1:1:2.5:15) was packed into drilled graphite rods (100 mm in length and 8 mm in diameter). After arc discharging, the soot was pre-extracted with acetone for two hours to remove non-fullerene products and further Soxhlet extracted for 20 h with carbon disulphide. The isolation of $LaSc_2N@C_{80}$ was performed by HPLC as described in Figure S1. Laser desorption time-of-flight mass-spectra were measured using Biflex III (Bruker, Germany).

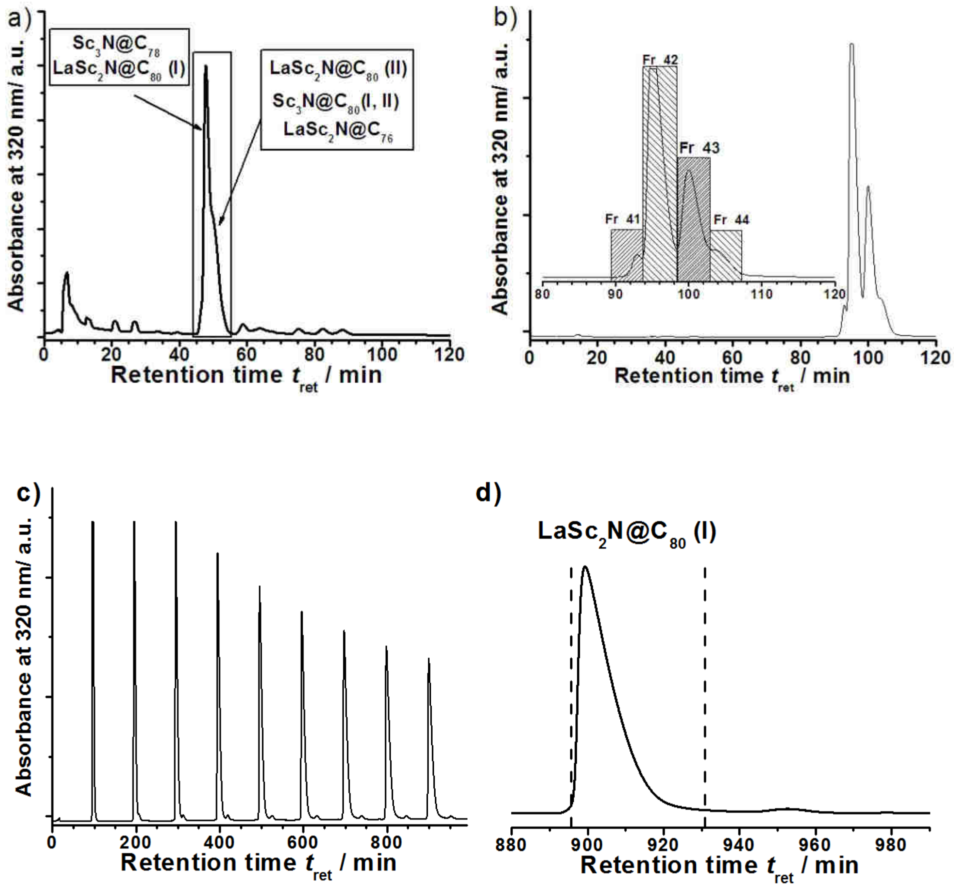

Figure S1 Chromatogram of $La_xSc_{3-x}N@C_{2n}$ (2n= 78-88) fullerene extract mixture (a 10×250 mm Buckyprep columns, flow rate 3.0 ml/min, injection volume 2 mL, toluene as mobile phase, 20 °C). (b) $LaSc_2N@C_{80}$ (I) was firstly enriched in fraction 42. (c) $LaSc_2N@C_{80}$ (I) was obtained by recycling HPLC after 9 cycles (10×250 mm Buckyprep column; flow rate 1.5 ml/min; injection volume is 4 ml; toluene as eluent, 20 ℃); The enlarged views of 9th cycle from (c) is shown in (d), (t_{ret}= 880 - 990 min).

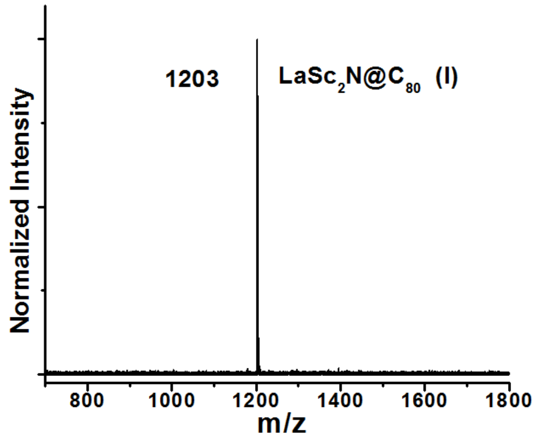

Figure S1 (continued). LDI-TOF Mass spectrum of **1La**

Synthesis of 1Pr

Pr-Sc NCF mixture was synthesized using the same procedure as described for 1La.

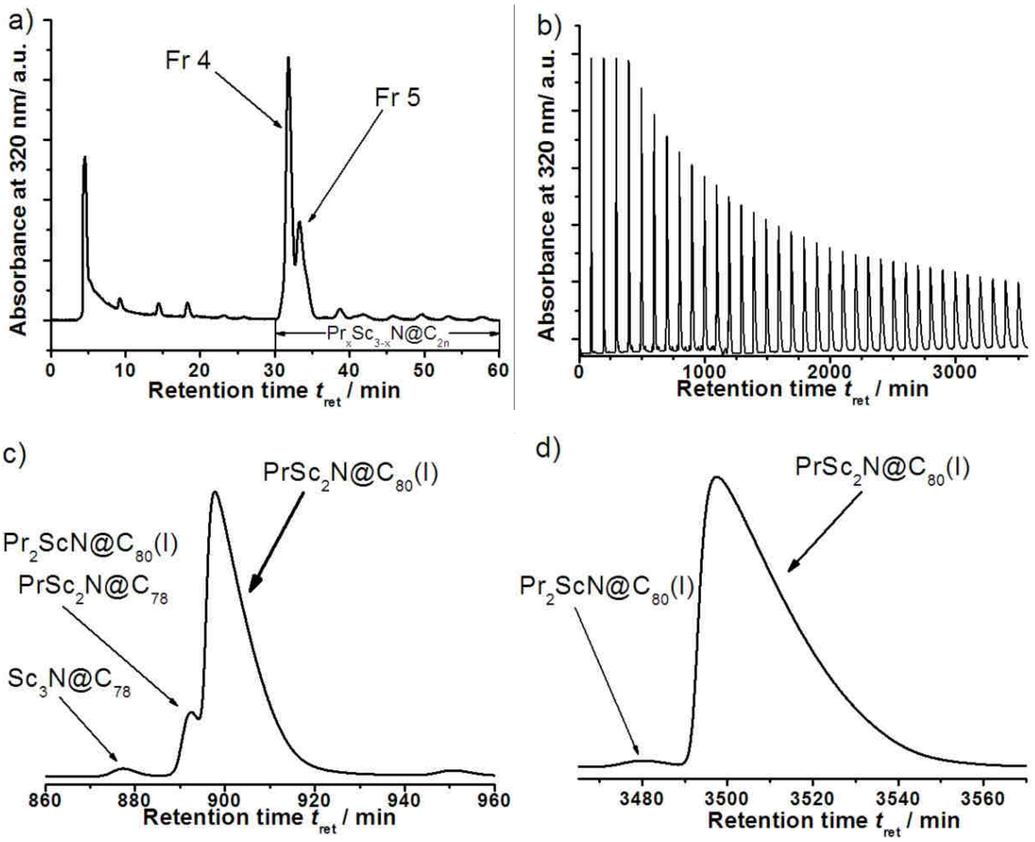

Figure S2. (a) Chromatogram of $Pr_xSc_{3-x}N@C_{2n}$ (2n= 78-88) fullerene extract mixture (combination of two 4.6×250 mm Buckyprep columns, flow rate 1.6 ml/min, injection volume 200 µL, toluene as mobile phase, 40 ℃). (b) $PrSc_2N@C_{80}$ (I) was firstly enriched in fraction 4. $PrSc_2N@C_{80}$ (I) was obtained by recycling HPLC after 35 cycles (10×250 mm Buckyprep column; flow rate 1.5 ml/min; injection volume is 5 ml; toluene as eluent); The enlarged views of 9th and 35th cycle from (b) are shown in (c) and (d) respectively, (t_{ret}= 860 - 960 min and t_{ret}= 3465 – 3670 min).

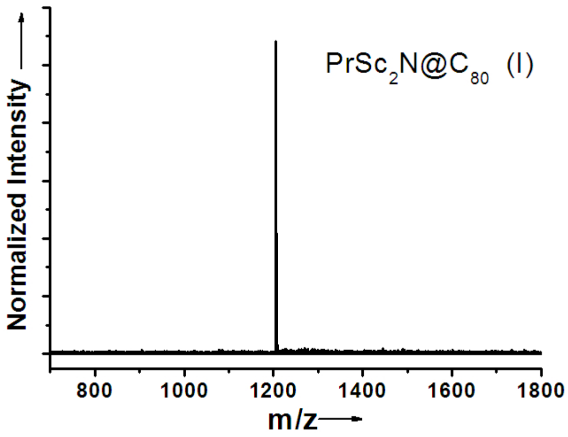

Figure S2 (continued). LDI-TOF Mass spectrum of **1Pr**

Synthesis of 1Tb

Tb-Sc NCF mixture was synthesized using Sc : Tb : C = 1 : 1 : 15 ratio and NH_3 (20 mbar) as a reactive gas.

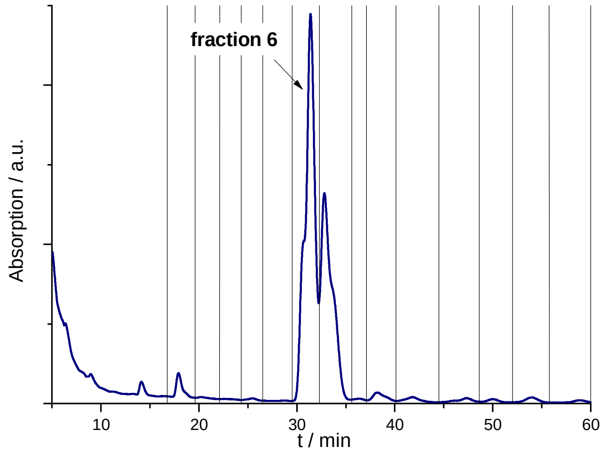

Figure S3a. Chromatogram of $Tb_xSc_{3-x}N@C_{2n}$ (2n= 78-88) fullerene extract mixture (combination of two 4.6×250 mm Buckyprep columns, flow rate 1.6 ml/min, injection volume 0.8 ml, toluene as mobile phase, 40 ℃).

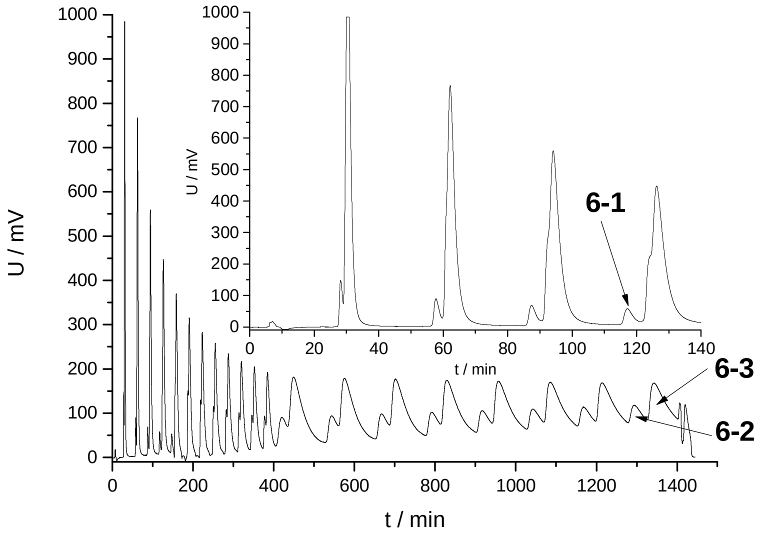

Figure S3b. Recycling-HPLC chromatography of the fraction 6, the inset shows cycles 1-4 (injection volume: 3,5 ml, flow rate: 3.0 ml/min for cycles 1 – 12 and 21;0.78 ml/min for cycles 13 – 20. Column: Buckyprep-M 10 mm x 250 mm, λ = 320 nm, temperature 23 °C, eluent: toluene). According to mass-spectral analysis, fraction **6-1** is $Sc_3N@C_{78}$, fraction **6-2** is mainly $Tb_2ScN@C_{80}$, and fraction **6-3** is $TbSc_2N@C_{80}$.

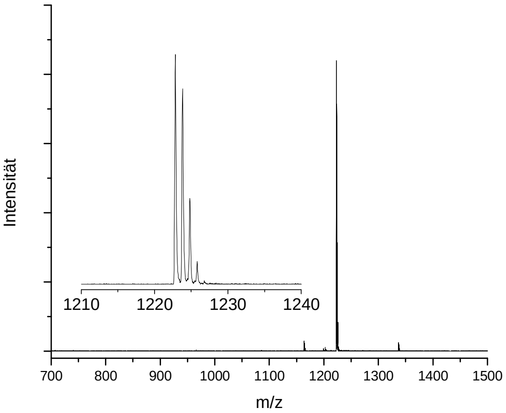

Figure S3c. Mass-spectrum of $TbSc_2N@C_{80}$

Synthesis of 1Tm

Tm-Sc NCF mixture was synthesized using the same procedure as described for 1La.

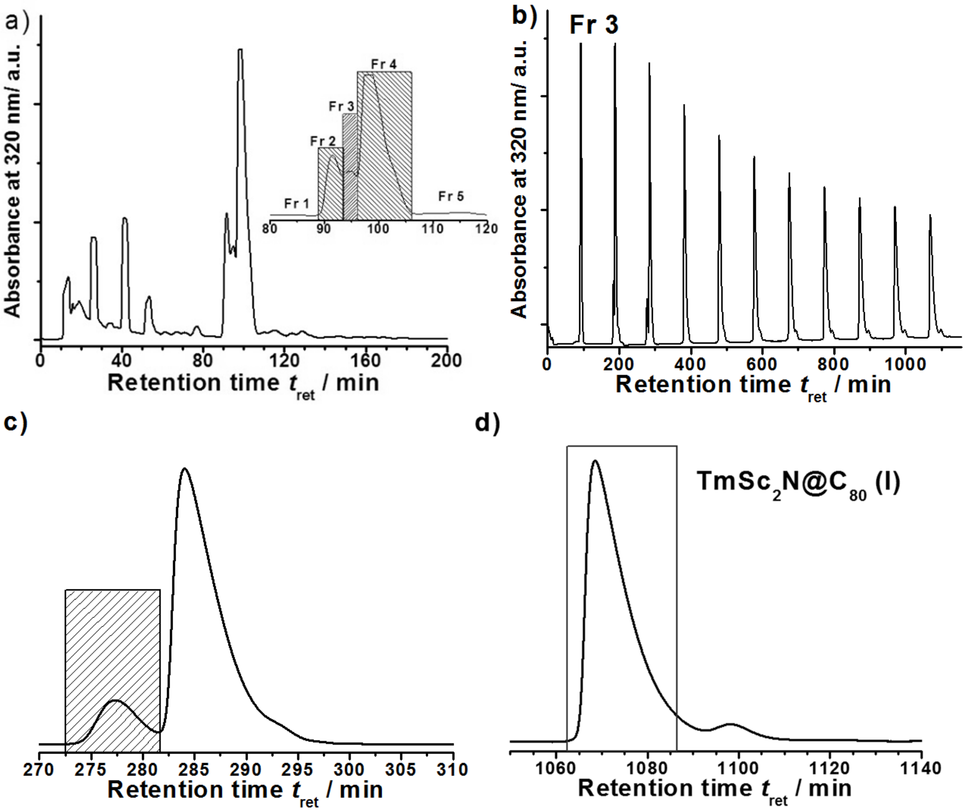

Figure S4. (a) Chromatogram of $Tm_xSc_{3-x}N@C_{2n}$ (2n= 78-88) fullerene extract mixture (a 10×250 mm Buckyprep columns, flow rate 3.0 ml/min, injection volume 2 mL, toluene as mobile phase, 20 ℃). (b) $TmSc_2N@C_{80}$ (I) was firstly enriched in fraction 3. $TmSc_2N@C_{80}$ (I) was obtained by recycling HPLC after 11 cycles (10×250 mm Buckyprep column; flow rate 1.5 ml/min; injection volume is 3 ml; toluene as eluent; 20 ℃); The enlarged views of 3rd (to remove $Tm_2ScN@C_{80}$ (I)) and 11st cycle (to remove $Sc_3N@C_{80}$ (I)) from (b) are shown in (c) and (d) respectively, (t_{ret}= 270 - 310 min and t_{ret}= 1050 – 1140 min).

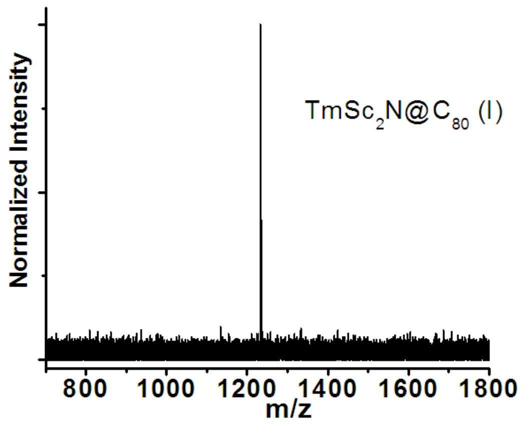

Figure S4 (continued). LDI-TOF Mass spectrum of **1Tm**

Synthesis of 1Er

Er-Sc NCF mixture was synthesized using the same procedure as described for 1La.

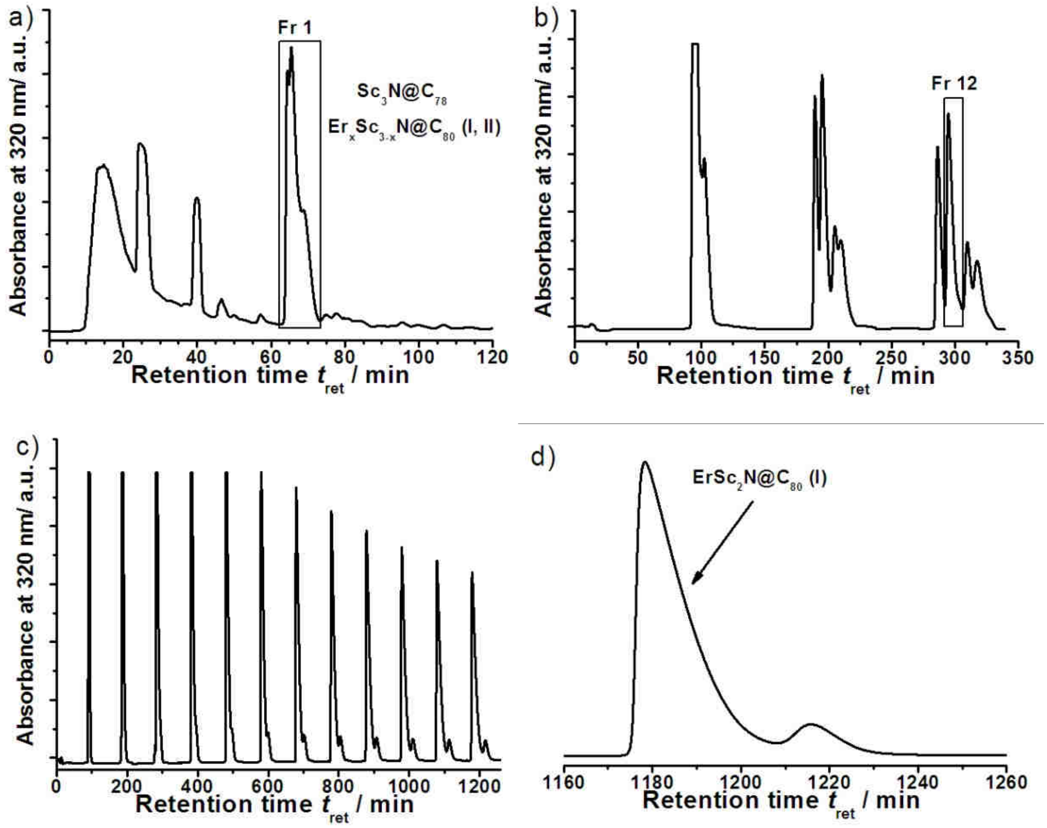

Figure S5. (a) Chromatogram of $Er_xSc_{3-x}N@C_{2n}$ (2n= 78-88) fullerene extract mixture which synthesized by the "SOS" method (a 10×250 mm Buckyprep columns, flow rate 2.0 ml/min, injection volume 2 mL, toluene as mobile phase, 20 ℃). (b) $ErSc_2N@C_{80}$ (I) was firstly enriched in fraction 1. $ErSc_2N@C_{80}$ (I) was obtained by recycling HPLC after 12 cycles (10×250 mm Buckyprep column; flow rate 1.5 ml/min; injection volume is 3 ml; toluene as eluent; 20 ℃); The enlarged views of 12th cycle (to remove $Sc_3N@C_{80}$ (I)) from (c) is shown in (d), (t_{ret}= 1160 – 1260 min).

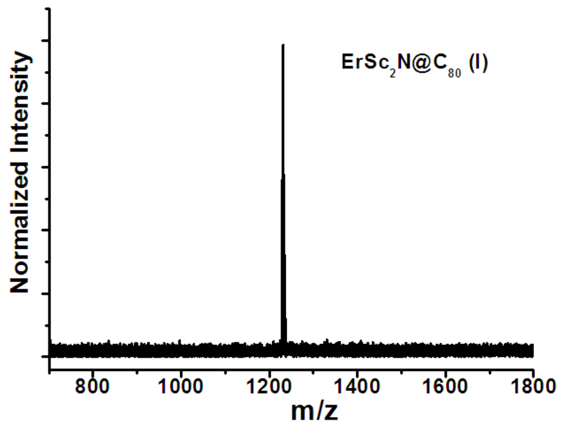

Figure S5 (continued). LDI-TOF Mass spectrum of **1Er**

Estimation of δ^{dia} values

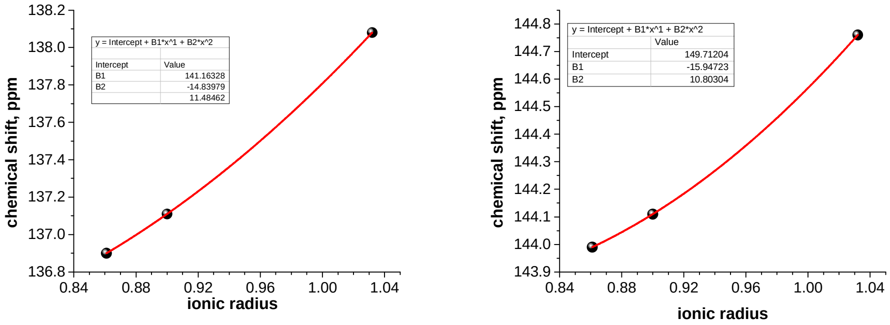

Figure S6. Fitting of diamagnetic ^{13}C chemical shifts in **1Lu**, **1Y**, and **1La** (THJ – left, PHHJ - right) by quadratic functions of ionic radius.

Relaxation times

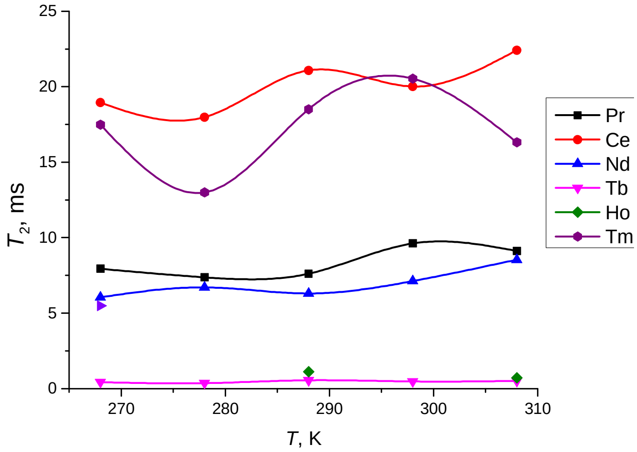

Figure S7. Temperature dependence of T_2 relaxation time for PHHJ carbons estimated from the peak width.

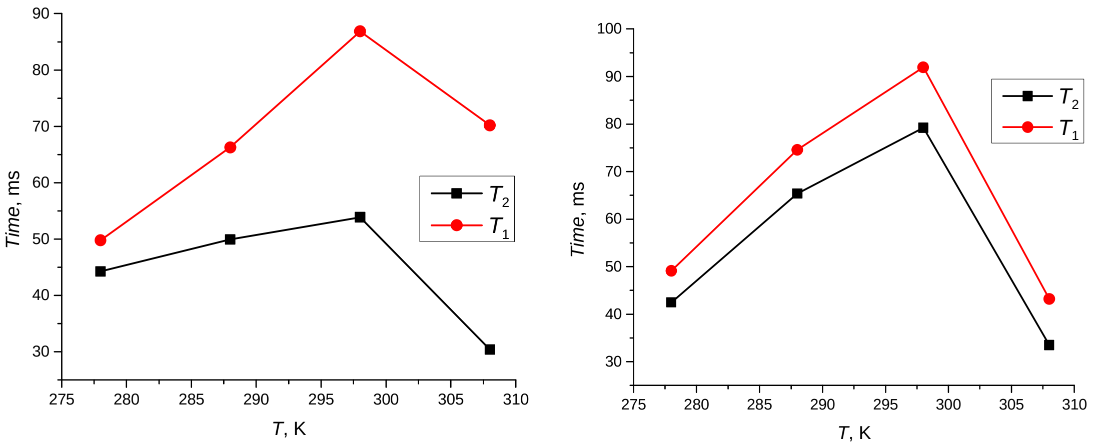

Figure S8. T_1 and T_2 relaxation times of PHHJ (left) and THJ (right) carbons in $TmSc_2N@C_{80}$ measured by inversion recovery method (T_1) and Carr-Purcell-Meiboom-Gill pulse sequence (T_2).

Selected T_1 times (ms) measured by inversion recovery method at different temperatures:

1Nd, PHHJ: 6.5 (278), 8.7 (288), 14.5 (298), 10.5 (308)

1Pr, PHHJ: 9.3 (298), 11.4 (308)

Peak width versus paramagnetic shift

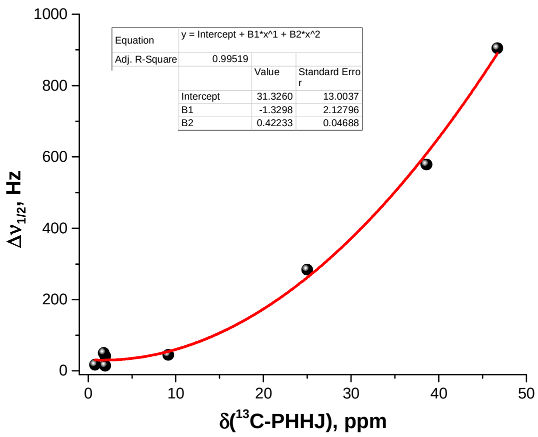

Figure S9. Correlation between peak width and absolute paramagnetic shift for ^{13}C-PHHJ carbons

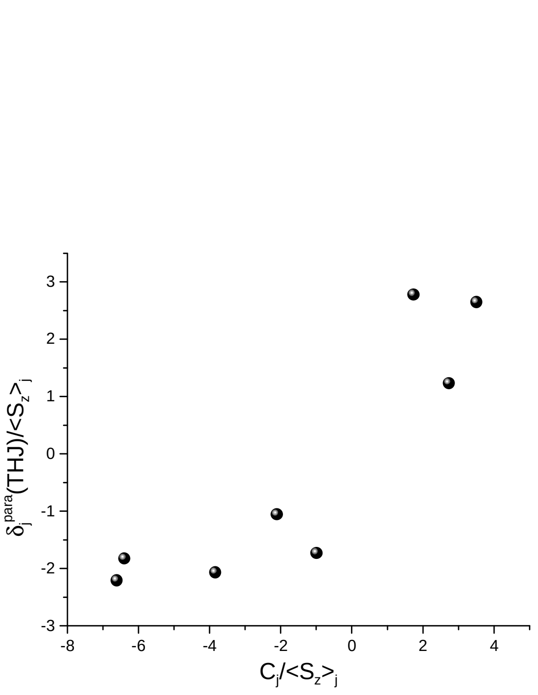

Figure S10. Correlations between $\delta_{ij}^{para}/\langle S_z \rangle_j$ and $C_j/\langle S_z \rangle_j$ for ^{13}C-THJ used to distinguish contact and pseudocontact contributions in **1M** molecules by Reilley's approach. The lack of linear correlation does not allow us to use Reilley's approach for ^{13}C-THJ carbons.

Table S1. Diamagnetic chemical shifts estimated from T^{-1} and T^{-2} temperature dependences of chemical shifts[a]

	^{13}C-PHHJ			^{13}C-THJ			^{45}Sc [c]	
**1M**	δ^{dia} (exp)[b]	δ^{dia} (T^{-1})	δ^{dia} (T^{-2})	δ^{dia} (exp)[b]	δ^{dia} (T^{-1})	δ^{dia} (T^{-2})	δ^{dia} (T^{-1})	δ^{dia} (T^{-2})
Ce	144.63	147.9	145.3	137.89	139.8	137.8	135 [d]	190 [d]
Pr	144.51	147.7	145.1	137.73	138.3	135.0	160	308
Nd	144.47	144.5	143.6	137.67	140.6	135.2	255	308
Tb	144.20	167.8	136.7	137.25	152.3	125.1	-301	327
Dy	144.15	171.3	134.6	137.18	n/a	n/a	-188	850
Ho	144.11	n/a	n/a	137.12	n/a	n/a	-531 [e]	271 [e]
Er	144.08	138.01	145.6	137.05	137.8	142.0	536	149
Tm	144.04	147.47	146.2	137.00	130.87	141.5	246	120

[a] Estimation of δ^{dia} from temperature dependence of chemical shifts is based the assumption that either contact or pseudocontact terms have predominant contribution; since these terms are expected to have T^{-1} or T^{-2} dependences, respectively, linearization of the equations $\delta(T) = \delta^{dia} + const \cdot T^{-1}$ or $\delta(T) = \delta^{dia} + const \cdot T^{-2}$ gives δ^{dia} as an intercept; [b] diamagnetic shifts were estimated from the polynomial fit of the δ(**1M**)–*vs*–R(M^{3+}) data for **1Y**, **1La**, and **1Lu**; [c] diamagnetic ^{45}Sc chemical shift is ~ 200 ppm; [d] from Wang *et al., J. Am. Chem. Soc.* **2006**, *128*, 8884-8889; from Zhang *et al., Chem. Eur. J.* **2012**, *18*, 9691 – 9698;

Table S2. Metal-nitrogen bond distances in DFT-optimized $MSc_2N@C_{80}$ molecules (*d*, Å) and QTAIM-computed atomic charges (*q*) used in point-charge calculations of the ligand field splitting

	d				***q***							
	**M-N**	**ScI-N**	**ScII-N**	**M-C1**	**M**	**N**	**ScI**	**ScII**	**C1**	**C2**	**C3**	**C4**
**La**	2.241	1.927	1.931	2.542	1.873	-1.654	1.710	1.704	-0.094	-0.094	-0.063	-0.087
**Ce**	2.233	1.932	1.935	2.524	1.865	-1.655	1.712	1.706	-0.095	-0.095	-0.064	-0.088
**Pr**	2.225	1.936	1.939	2.508	1.858	-1.656	1.713	1.707	-0.096	-0.096	-0.065	-0.088
**Nd**	2.217	1.939	1.942	2.493	1.851	-1.657	1.714	1.708	-0.098	-0.096	-0.066	-0.089
**Tb**	2.179	1.958	1.961	2.428	1.932	-1.704	1.723	1.718	-0.106	-0.107	-0.078	-0.102
**Dy**	2.173	1.961	1.964	2.416	1.928	-1.705	1.724	1.719	-0.107	-0.108	-0.080	-0.102
**Ho**	2.163	1.966	1.968	2.405	1.924	-1.706	1.725	1.720	-0.108	-0.109	-0.080	-0.103
**Er**	2.155	1.970	1.972	2.394	1.920	-1.706	1.726	1.721	-0.109	-0.109	-0.081	-0.104
**Tm**	2.147	1.974	1.976	2.384	1.916	-1.707	1.727	1.722	-0.110	-0.110	-0.082	-0.104
**Lu**	2.133	1.978	1.981	2.384	1.918	-1.709	1.730	1.726	-0.111	-0.111	-0.083	-0.105

See Figure 1 in the manuscript for the numbering of carbon atoms

Calculations of the ligand field splitting

Ligand filed splitting was computed using QTAIM charges listed in Table S2. To verify reliability of such calculations, we compared the energy levels with those computed ab initio at the CASSCF level from [Chibotaru *et al. J. Phys. Chem. Lett.* **2013**, *4*, 3565]. We used the set of values computed with extended basis set ("basis set 2" in original publication) and the same B3LYP-optimized coordinates as used in this work (in their computations Chibotaru *et al.* used Cartesian coordinates provided by our group).

In the point charge model, atomic (or other) charges are used to model the ligand field. Unfortunately, "atomic charge" is not well defined parameter in quantum chemistry – there are dozens of ways how atomic charges can be computed, with very different results. We prefer to use Bader (aka QTAIM) charges which are obtained by integration of the electron density over atomic basins. As such, QTAIM charges are stable with respect to the variation of the basis set and wavefunction method. Therefore, the scaling factor we used to reduce the charges has no solid physical meaning and should be considered merely as a fitting parameter. Namely, when QTAIM charges are scaled by 0.754 and used in the point charge model, good agreement with ab initio computed LF splitting is obtained (Table S3). It is known that QTAIM usually provides more ionic description of the systems than other approaches (such as Mulliken, Hirschfeld, etc). Thus, the use of, say, Mulliken charges would probably require completely different scaling factor.

Point charge model enables simple evaluation of the importance of different parts of the molecule in the ligand filed. To analyze this, we computed LF levels with several structural models. First, we used N, Sc, and four carbon atoms. The only N and Sc ions were considered. Finally, only the nitride ion was left. Atomic charges were the same in all calculations. The LF levels are listed in Table S3. It can be seen that Dy-N system overestimates the splitting. Yet, this is fairly uniform overestimation, and the qualitative description is similar to that of the more realistic models. Addition of Sc ions overcorrects the level energies: they are now smaller than in the full model. Anyway, it can be seen that the effect of the nitride is considerably larger than that of Sc ions. Addition of 4 closest carbon atoms has noticeable but not dramatic effect: the values are slightly increased by ca 12% in comparison to the N+2Sc model. Thus, we can conclude that the nitride ions has the largest effect on the ligand field, and hence the LF is almost uniaxial; energy levels computed only with N are 23-25% high in energy that the model including Sc and carbons. Sc reduces the symmetry, and down-scales the energy levels by ca 40%; the values for N+2Sc model are 11-12% smaller than the model with carbon atoms. Thus, the large anisotropy is the result of the highly charged nitride ion located close to the lanthanide.

Table S3. LF levels (cm^{-1}) in $DySc_2N@C_{80}$ computed with different charge models

CASSCF[a]	PCM		
	N+2Sc+4C	N+2Sc	N
0	0	0	0
372	382	340	469
729	699	621	860
1009	944	837	1168
1180	1121	990	1396
1268	1236	1086	1555
1338	1309	1153	1653
1401	1401	1240	1701

[a] from [Chibotaru *et al. J. Phys. Chem. Lett.* **2013**, *4*, 3565]

Table S4. Ligand field splitting levels (cm^{-1}) in 1M molecules, their degeneracies, and description in terms of $|\pm m_J\rangle$ basis functions (contributions less than 0.05 are not shown)

**Ce**		
0.0	2	0.99 $\vert \pm 5/2\rangle$
1074.6	2	0.98 $\vert \pm 3/2\rangle$
1914.6	2	0.99 $\vert \pm 1/2\rangle$
**Pr**		
0.0	2	1.00 $\vert \pm 4\rangle$
849.3	1	0.99 $\vert \pm 3\rangle$
850.2	1	0.99 $\vert \pm 3\rangle$
1358.8	1	0.90 $\vert \pm 2\rangle$ + 0.09 $\vert 0\rangle$
1389.3	1	0.99 $\vert \pm 2\rangle$
1544.3	1	0.99 $\vert \pm 1\rangle$
1679.4	1	0.93 $\vert \pm 1\rangle$
1686.8	1	0.85 $\vert 0\rangle$ + 0.08 $\vert \pm 2\rangle$ + 0.06 $\vert \pm 1\rangle$
**Nd**		
0.0	2	1.00 $\vert \pm 9/2\rangle$
363.8	2	0.99 $\vert \pm 7/2\rangle$
497.0	2	0.97 $\vert \pm 5/2\rangle$
591.8	2	0.90 $\vert \pm 3/2\rangle$ + 0.09 $\vert \pm 1/2\rangle$
674.7	2	0.89 $\vert \pm 1/2\rangle$ + 0.09 $\vert \pm 3/2\rangle$
**Tb**		
0.0	2	1.00 $\vert \pm 6\rangle$
384.3	2	1.00 $\vert \pm 5\rangle$
729.6	1	0.99 $\vert \pm 4\rangle$
729.7	1	0.99 $\vert \pm 4\rangle$
1020.5	1	0.97 $\vert \pm 3\rangle$
1022.4	1	0.98 $\vert \pm 3\rangle$
1228.0	1	0.88 $\vert \pm 2\rangle$ + 0.11 $\vert 0\rangle$
1254.7	1	0.99 $\vert \pm 2\rangle$
1345.4	1	0.97 $\vert \pm 1\rangle$
1454.1	1	0.98 $\vert \pm 1\rangle$
1468.4	1	0.89 $\vert 0\rangle$ + 0.11 $\vert \pm 2\rangle$
**Dy**		
0.0	2	1.00 $\vert \pm 15/2\rangle$
382.3	2	0.99 $\vert \pm 13/2\rangle$
698.8	2	0.98 $\vert \pm 11/2\rangle$
943.9	2	0.97 $\vert \pm 9/2\rangle$
1120.9	2	0.93 $\vert \pm 7/2\rangle$ + 0.05 $\vert \pm 3/2\rangle$
1235.7	2	0.79 $\vert \pm 5/2\rangle$ + 0.15 $\vert \pm 1/2\rangle$
1309.5	2	0.64 $\vert \pm 3/2\rangle$ + 0.19 $\vert \pm 1/2\rangle$ + 0.13 $\vert \pm 5/2\rangle$
1400.8	2	0.66 $\vert \pm 1/2\rangle$ + 0.27 $\vert \pm 3/2\rangle$ + 0.06 $\vert \pm 5/2\rangle$

**Ho**		
0.0	2	1.00 $\lvert \pm 8\rangle$
193.3	2	0.99 $\lvert \pm 7\rangle$
306.9	2	0.99 $\lvert \pm 6\rangle$
381.9	2	0.97 $\lvert \pm 5\rangle$
438.6	1	0.95 $\lvert \pm 4\rangle$
438.9	1	0.95 $\lvert \pm 4\rangle$
483.1	1	0.87 $\lvert \pm 3\rangle$ + 0.1 $\lvert \pm 1\rangle$
486.2	1	0.93 $\lvert \pm 3\rangle$
512.8	1	0.73 $\lvert \pm 2\rangle$ + 0.23 $\lvert 0\rangle$
528.3	1	0.96 $\lvert \pm 2\rangle$
536.9	1	0.89 $\lvert \pm 1\rangle$ + 0.1 $\lvert \pm 3\rangle$
572.5	1	0.96 $\lvert \pm 1\rangle$
573.3	1	0.76 $\lvert 0\rangle$ + 0.23 $\lvert \pm 2\rangle$
**Er**		
0.0	2	0.72 $\lvert \pm 1/2\rangle$ + 0.23 $\lvert \pm 3/2\rangle$ + 0.05 $\lvert \pm 5/2\rangle$
37.0	2	0.72 $\lvert \pm 3/2\rangle$ + 0.19 $\lvert \pm 1/2\rangle$ + 0.06 $\lvert \pm 5/2\rangle$
69.6	2	0.87 $\lvert \pm 5/2\rangle$ + 0.09 $\lvert \pm 1/2\rangle$
115.4	2	0.95 $\lvert \pm 7/2\rangle$
173.8	2	0.97 $\lvert \pm 9/2\rangle$
249.9	2	0.99 $\lvert \pm 11/2\rangle$
362.9	2	0.99 $\lvert \pm 13/2\rangle$
554.1	2	1.00 $\lvert \pm 15/2\rangle$
**Tm**		
0.0	1	0.82 $\lvert 0\rangle$ + 0.18 $\lvert \pm 2\rangle$
3.5	1	0.97 $\lvert \pm 1\rangle$
86.1	1	0.95 $\lvert \pm 1\rangle$
127.5	1	0.98 $\lvert \pm 2\rangle$
155.0	1	0.81 $\lvert \pm 2\rangle$ + 0.17 $\lvert 0\rangle$
284.5	1	0.97 $\lvert \pm 3\rangle$
287.0	1	0.95 $\lvert \pm 3\rangle$
522.0	1	0.99 $\lvert \pm 4\rangle$
522.0	1	0.99 $\lvert \pm 4\rangle$
862.3	2	1.00 $\lvert \pm 5\rangle$
1298.4	2	1.00 $\lvert \pm 6\rangle$

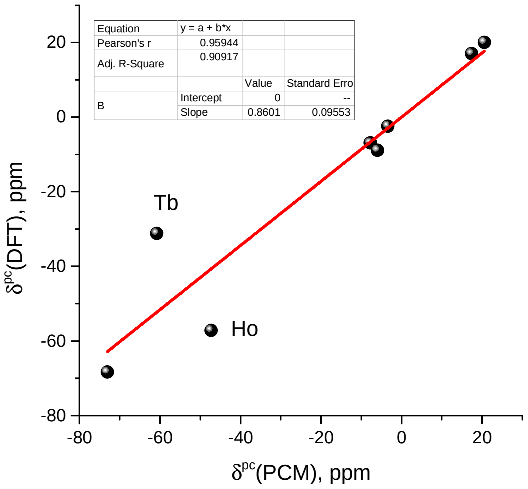

Figure S11. Correlation between computed and experimental chemical pseudocontact shifts for ^{13}C-THJ. Experimental $\delta^{pc}(^{13}C)$ shifts are obtained by subtracting δ^{con} values obtained by DFT calculations form experimental paramagnetic shifts.

DFT-optimized Cartesian Coordinates (Å) of $MSc_2N@C_{80}$-I_h molecules

$LaSc_2N@C_{80}$

C	-3.025783000	-1.268402000	2.508112000
C	2.564336000	-1.998260000	2.515854000
C	0.418363000	3.227554000	2.502149000
C	-3.348695000	-2.108412000	1.381688000
C	3.386887000	-1.835524000	1.363734000
C	-0.140404000	3.962470000	1.399985000
C	-2.523595000	-3.289905000	1.131595000
C	3.967395000	-0.531285000	1.089112000
C	-1.584871000	3.832537000	1.150139000
C	-3.904487000	-1.430196000	0.238184000
C	3.111416000	-2.672512000	0.244657000
C	0.730600000	4.084451000	0.247437000
C	-3.250017000	0.162952000	2.507287000
C	1.444416000	-2.913882000	2.507943000
C	1.776460000	2.712817000	2.504337000
C	-0.273317000	-3.985408000	1.100041000
C	3.549724000	1.763628000	1.081107000
C	-3.250161000	2.155719000	1.083693000
C	-3.735083000	0.819984000	1.349423000
C	1.123476000	-3.691066000	1.360667000
C	2.607715000	2.833980000	1.358103000
C	-4.094562000	0.007233000	0.233444000
C	2.005161000	-3.590412000	0.240665000
C	2.080355000	3.545822000	0.242062000
C	-3.584229000	-1.841083000	-1.121888000
C	3.303024000	-2.170757000	-1.100976000
C	0.221236000	3.980112000	-1.112378000
C	-2.710759000	-2.963400000	-1.395772000
C	3.878258000	-0.878932000	-1.387634000
C	-1.182569000	3.759131000	-1.387113000
C	-1.815535000	-2.764116000	-2.514358000
C	3.278409000	-0.202141000	-2.515011000
C	-1.464445000	2.902062000	-2.506082000
C	-3.576367000	-0.635011000	-1.936865000
C	2.289762000	-2.797184000	-1.926172000
C	1.260049000	3.383807000	-1.926707000
C	0.350495000	-2.380238000	-3.343781000
C	1.862968000	1.482343000	-3.336899000
C	-2.230868000	0.852930000	-3.357042000
C	-2.688733000	-0.469714000	-3.044394000
C	1.721363000	-2.110746000	-3.034864000
C	0.950073000	2.541261000	-3.037776000
C	-1.829330000	-1.569849000	-3.340624000
C	2.248077000	-0.813414000	-3.330559000

C	-0.428299000	2.336665000	-3.338084000
C	-0.797964000	-4.137414000	-0.241811000
C	4.019996000	1.409743000	-0.251344000
C	-3.104065000	2.673063000	-0.246874000
C	0.098739000	-3.893309000	-1.363190000
C	3.332293000	2.047572000	-1.370517000
C	-3.380713000	1.799485000	-1.369190000
C	-3.906518000	0.490448000	-1.104825000
C	1.495154000	-3.672158000	-1.102912000
C	2.410574000	3.130540000	-1.097037000
C	-0.447433000	-3.249990000	-2.513516000
C	3.021945000	1.231693000	-2.508790000
C	-2.576828000	1.969712000	-2.523716000
C	-0.502632000	-1.340937000	-3.845615000
C	1.391638000	0.222525000	-3.836453000
C	-0.898501000	1.076460000	-3.850599000
C	-0.003537000	-0.014858000	-4.064917000
C	-2.208563000	-3.734065000	-0.245808000
C	4.312941000	-0.049679000	-0.251451000
C	-2.100229000	3.729302000	-0.244124000
C	0.408797000	-2.361581000	3.340849000
C	1.806918000	1.538527000	3.335766000
C	-2.254620000	0.789578000	3.340135000
C	-0.972487000	-2.576272000	3.042748000
C	2.661187000	0.437565000	3.031175000
C	-1.734440000	2.088995000	3.043776000
C	-1.317583000	-3.453705000	1.936493000
C	3.542853000	0.582806000	1.897499000
C	-2.298431000	2.822575000	1.928555000
C	-1.871622000	-1.510643000	3.342460000
C	2.208776000	-0.876350000	3.345803000
C	-0.368207000	2.347727000	3.346732000
C	-1.401282000	-0.248005000	3.853551000
C	0.883112000	-1.101490000	3.857875000
C	0.486463000	1.317682000	3.860708000
C	-0.009929000	-0.010528000	4.073957000
N	0.230321000	0.349965000	0.034285000
La	-1.064400000	-1.478445000	0.008174000
Sc	2.121156000	0.210332000	-0.312816000
Sc	-0.669662000	2.051620000	0.185912000

$CeSc_2N@C_{80}$

C	-3.024279000	-1.266441000	2.508912000
C	2.565467000	-1.998261000	2.517415000
C	0.418642000	3.226577000	2.502952000
C	-3.345310000	-2.104726000	1.381401000
C	3.387448000	-1.835853000	1.364653000
C	-0.140115000	3.958714000	1.399294000
C	-2.520678000	-3.285626000	1.131141000
C	3.967102000	-0.531657000	1.090088000
C	-1.583560000	3.829387000	1.149399000
C	-3.901247000	-1.427260000	0.238152000
C	3.112550000	-2.672595000	0.244939000
C	0.730052000	4.081241000	0.247241000
C	-3.249813000	0.164257000	2.508341000
C	1.445812000	-2.913617000	2.509428000
C	1.776620000	2.712546000	2.505511000
C	-0.271081000	-3.979749000	1.099238000
C	3.547730000	1.762518000	1.081595000
C	-3.250649000	2.155969000	1.084119000
C	-3.735686000	0.820843000	1.350273000
C	1.125637000	-3.689505000	1.361167000
C	2.606893000	2.833069000	1.358576000
C	-4.094462000	0.009136000	0.233373000
C	2.006924000	-3.590593000	0.240846000
C	2.079639000	3.544142000	0.242080000
C	-3.581169000	-1.838126000	-1.121615000
C	3.303277000	-2.170700000	-1.101000000
C	0.220944000	3.977810000	-1.112305000
C	-2.708312000	-2.960235000	-1.395629000
C	3.876533000	-0.878453000	-1.387288000
C	-1.182233000	3.757284000	-1.387045000
C	-1.813486000	-2.761029000	-2.513784000
C	3.276742000	-0.202113000	-2.514672000
C	-1.464585000	2.902110000	-2.507089000
C	-3.575871000	-0.633087000	-1.937492000
C	2.290667000	-2.796971000	-1.926710000
C	1.259760000	3.383214000	-1.927257000
C	0.351412000	-2.379510000	-3.345523000
C	1.862611000	1.482381000	-3.338221000
C	-2.231091000	0.853923000	-3.358574000
C	-2.688525000	-0.468396000	-3.045419000
C	1.722218000	-2.110726000	-3.036020000
C	0.949834000	2.541276000	-3.038776000
C	-1.828479000	-1.568165000	-3.341487000
C	2.248043000	-0.813378000	-3.331859000
C	-0.428489000	2.337087000	-3.339411000
C	-0.795822000	-4.132293000	-0.241981000
C	4.015419000	1.407846000	-0.250859000

C	-3.104355000	2.672792000	-0.246863000
C	0.100614000	-3.890198000	-1.363329000
C	3.329051000	2.045718000	-1.369830000
C	-3.381242000	1.800399000	-1.369921000
C	-3.907397000	0.491925000	-1.105466000
C	1.496856000	-3.671841000	-1.103026000
C	2.409610000	3.129676000	-1.097150000
C	-0.445713000	-3.247446000	-2.513655000
C	3.019936000	1.231078000	-2.508601000
C	-2.577093000	1.970340000	-2.524618000
C	-0.502158000	-1.340210000	-3.847556000
C	1.391748000	0.222676000	-3.838386000
C	-0.898638000	1.077119000	-3.852281000
C	-0.003429000	-0.014195000	-4.067066000
C	-2.206313000	-3.730612000	-0.245900000
C	4.310035000	-0.049911000	-0.250988000
C	-2.099081000	3.727134000	-0.244062000
C	0.410112000	-2.361069000	3.342486000
C	1.807517000	1.538687000	3.337622000
C	-2.254603000	0.790480000	3.341840000
C	-0.970914000	-2.573808000	3.043733000
C	2.661701000	0.437627000	3.032878000
C	-1.734232000	2.089254000	3.044855000
C	-1.314936000	-3.448093000	1.935303000
C	3.542473000	0.582321000	1.898574000
C	-2.298107000	2.821672000	1.928792000
C	-1.870669000	-1.509088000	3.343973000
C	2.209781000	-0.876268000	3.347660000
C	-0.367907000	2.347691000	3.347977000
C	-1.400961000	-0.247009000	3.855673000
C	0.884105000	-1.101153000	3.859796000
C	0.486993000	1.317947000	3.862496000
C	-0.009313000	-0.010178000	4.075942000
N	0.217107000	0.332588000	0.025566000
Ce	-1.073861000	-1.488673000	0.004319000
Sc	2.115194000	0.201747000	-0.307781000
Sc	-0.673818000	2.042712000	0.184256000

$PrSc_2N@C_{80}$

C	-3.022834000	-1.264737000	2.509539000
C	2.566511000	-1.998081000	2.518816000
C	0.418989000	3.225745000	2.503771000
C	-3.342670000	-2.101706000	1.381293000
C	3.388100000	-1.835890000	1.365522000
C	-0.139717000	3.955031000	1.398561000
C	-2.518134000	-3.281867000	1.130846000
C	3.967570000	-0.531960000	1.091239000
C	-1.582080000	3.825967000	1.148525000
C	-3.898872000	-1.424855000	0.238167000
C	3.113423000	-2.672243000	0.245131000
C	0.729634000	4.078554000	0.247013000
C	-3.249535000	0.165354000	2.509282000
C	1.447141000	-2.913175000	2.510669000
C	1.776745000	2.712300000	2.506477000
C	-0.268914000	-3.974235000	1.098489000
C	3.545534000	1.761413000	1.082088000
C	-3.250769000	2.155863000	1.084346000
C	-3.736120000	0.821454000	1.351007000
C	1.127616000	-3.687744000	1.361492000
C	2.606122000	2.832394000	1.358941000
C	-4.094446000	0.010678000	0.233360000
C	2.008510000	-3.590472000	0.240872000
C	2.079149000	3.542963000	0.242015000
C	-3.578818000	-1.835604000	-1.121464000
C	3.303577000	-2.170395000	-1.101132000
C	0.220678000	3.976121000	-1.112344000
C	-2.706084000	-2.957291000	-1.395502000
C	3.875312000	-0.877887000	-1.387152000
C	-1.181984000	3.755989000	-1.387103000
C	-1.811400000	-2.757987000	-2.513072000
C	3.274983000	-0.201985000	-2.514202000
C	-1.464693000	2.902179000	-2.507881000
C	-3.575314000	-0.631400000	-1.937930000
C	2.291614000	-2.796659000	-1.927348000
C	1.259534000	3.382978000	-1.927843000
C	0.352313000	-2.378812000	-3.347283000
C	1.862256000	1.482547000	-3.339534000
C	-2.231219000	0.854811000	-3.359894000
C	-2.688240000	-0.467206000	-3.046256000
C	1.723036000	-2.110620000	-3.037231000
C	0.949670000	2.541469000	-3.039749000
C	-1.827533000	-1.566504000	-3.342159000
C	2.248013000	-0.813260000	-3.333143000
C	-0.428637000	2.337546000	-3.340556000
C	-0.793646000	-4.126837000	-0.242182000
C	4.010140000	1.405759000	-0.250341000

C	-3.104429000	2.672378000	-0.246926000
C	0.102476000	-3.887056000	-1.363575000
C	3.325149000	2.043740000	-1.368936000
C	-3.381654000	1.801057000	-1.370555000
C	-3.908222000	0.493140000	-1.105971000
C	1.498548000	-3.671396000	-1.103263000
C	2.408730000	3.129352000	-1.097325000
C	-0.444015000	-3.245130000	-2.514003000
C	3.017659000	1.230516000	-2.508269000
C	-2.577266000	1.970816000	-2.525356000
C	-0.501660000	-1.339468000	-3.849421000
C	1.391916000	0.222914000	-3.840353000
C	-0.898726000	1.077782000	-3.853787000
C	-0.003275000	-0.013520000	-4.069047000
C	-2.204064000	-3.727153000	-0.245991000
C	4.307633000	-0.050030000	-0.250593000
C	-2.097985000	3.725126000	-0.244078000
C	0.411324000	-2.360476000	3.343840000
C	1.808130000	1.538853000	3.339280000
C	-2.254519000	0.791201000	3.343377000
C	-0.969480000	-2.571489000	3.044610000
C	2.662312000	0.437732000	3.034564000
C	-1.733832000	2.089312000	3.045788000
C	-1.312534000	-3.442970000	1.934330000
C	3.542372000	0.581886000	1.899729000
C	-2.297386000	2.820342000	1.928751000
C	-1.869888000	-1.507762000	3.345522000
C	2.210780000	-0.876140000	3.349508000
C	-0.367554000	2.347767000	3.349300000
C	-1.400665000	-0.246139000	3.857655000
C	0.885022000	-1.100766000	3.861484000
C	0.487550000	1.318256000	3.864215000
C	-0.008725000	-0.009791000	4.077713000
N	0.205027000	0.315999000	0.017840000
Pr	-1.084997000	-1.496195000	0.001888000
Sc	2.109846000	0.189013000	-0.301244000
Sc	-0.676982000	2.034693000	0.180526000

$NdSc_2N@C_{80}$

C	-3.021490000	-1.262907000	2.510424000
C	2.567416000	-1.998015000	2.520182000
C	0.419208000	3.224896000	2.504521000
C	-3.339280000	-2.098103000	1.380955000
C	3.388495000	-1.836096000	1.366336000
C	-0.139458000	3.951611000	1.397908000
C	-2.515296000	-3.277725000	1.130308000
C	3.967286000	-0.532255000	1.092061000
C	-1.580956000	3.823149000	1.147885000
C	-3.895798000	-1.422003000	0.238103000
C	3.114258000	-2.672111000	0.245317000
C	0.729173000	4.075617000	0.246819000
C	-3.249355000	0.166554000	2.510296000
C	1.448255000	-2.912870000	2.511975000
C	1.776814000	2.712071000	2.507450000
C	-0.266947000	-3.969254000	1.097747000
C	3.543607000	1.760417000	1.082438000
C	-3.251269000	2.156118000	1.084797000
C	-3.736705000	0.822235000	1.351819000
C	1.129430000	-3.686266000	1.361906000
C	2.605340000	2.831643000	1.359313000
C	-4.094420000	0.012464000	0.233305000
C	2.009902000	-3.590432000	0.240980000
C	2.078625000	3.541609000	0.241977000
C	-3.575978000	-1.832844000	-1.121261000
C	3.303771000	-2.170259000	-1.101243000
C	0.220442000	3.973962000	-1.112288000
C	-2.703919000	-2.954446000	-1.395424000
C	3.873927000	-0.877457000	-1.387027000
C	-1.181656000	3.754192000	-1.386960000
C	-1.809660000	-2.755379000	-2.512770000
C	3.273573000	-0.201942000	-2.514034000
C	-1.464749000	2.902078000	-2.508668000
C	-3.574889000	-0.629642000	-1.938544000
C	2.292368000	-2.796432000	-1.927895000
C	1.259355000	3.382541000	-1.928410000
C	0.353095000	-2.378299000	-3.348976000
C	1.862004000	1.482601000	-3.340831000
C	-2.231374000	0.855634000	-3.361201000
C	-2.688088000	-0.466086000	-3.047224000
C	1.723736000	-2.110586000	-3.038307000
C	0.949528000	2.541511000	-3.040692000
C	-1.826813000	-1.565079000	-3.343008000
C	2.248015000	-0.813237000	-3.334393000
C	-0.428745000	2.337859000	-3.341692000
C	-0.791764000	-4.122511000	-0.242419000
C	4.005874000	1.404020000	-0.249981000

C	-3.104776000	2.672194000	-0.246870000
C	0.104071000	-3.884541000	-1.363860000
C	3.322233000	2.042175000	-1.368430000
C	-3.382158000	1.801876000	-1.371177000
C	-3.909075000	0.494506000	-1.106557000
C	1.499959000	-3.671059000	-1.103425000
C	2.408029000	3.128831000	-1.097543000
C	-0.442551000	-3.243167000	-2.514423000
C	3.015916000	1.230023000	-2.508245000
C	-2.577442000	1.971297000	-2.526083000
C	-0.501243000	-1.338900000	-3.851196000
C	1.392050000	0.223038000	-3.842163000
C	-0.898814000	1.078305000	-3.855236000
C	-0.003163000	-0.012996000	-4.070933000
C	-2.202001000	-3.723943000	-0.246131000
C	4.305071000	-0.050218000	-0.250272000
C	-2.097026000	3.723194000	-0.243999000
C	0.412394000	-2.359980000	3.345338000
C	1.808554000	1.538983000	3.340856000
C	-2.254561000	0.792061000	3.345019000
C	-0.968211000	-2.569325000	3.045676000
C	2.662661000	0.437800000	3.036009000
C	-1.733713000	2.089597000	3.046834000
C	-1.310172000	-3.437894000	1.933321000
C	3.541959000	0.581479000	1.900627000
C	-2.297167000	2.819575000	1.929019000
C	-1.869215000	-1.506434000	3.347191000
C	2.211592000	-0.876040000	3.351156000
C	-0.367356000	2.347774000	3.350445000
C	-1.400481000	-0.245239000	3.859751000
C	0.885853000	-1.100449000	3.863274000
C	0.487932000	1.318492000	3.865780000
C	-0.008257000	-0.009467000	4.079557000
N	0.193766000	0.300516000	0.012144000
Nd	-1.091681000	-1.505274000	-0.002157000
Sc	2.104722000	0.180307000	-0.296736000
Sc	-0.681462000	2.026454000	0.179244000

$TbSc_2N@C_{80}$

symmetry c1

C	-3.016457000	-1.256116000	2.514370000
C	2.570769000	-1.997385000	2.525509000
C	0.420220000	3.220982000	2.508278000
C	-3.324994000	-2.083025000	1.379225000
C	3.390562000	-1.836169000	1.369828000
C	-0.138174000	3.933624000	1.394013000
C	-2.503086000	-3.260207000	1.127954000
C	3.969419000	-0.533563000	1.096821000
C	-1.575347000	3.807662000	1.143646000
C	-3.883289000	-1.410114000	0.237806000
C	3.116616000	-2.670024000	0.245738000
C	0.727082000	4.062374000	0.245471000
C	-3.248685000	0.170777000	2.514096000
C	1.452524000	-2.911413000	2.516738000
C	1.776656000	2.710756000	2.511177000
C	-0.259000000	-3.950348000	1.095022000
C	3.533721000	1.755644000	1.084089000
C	-3.253226000	2.155803000	1.085784000
C	-3.739642000	0.824597000	1.354801000
C	1.136600000	-3.680630000	1.363433000
C	2.601531000	2.828767000	1.360536000
C	-4.096006000	0.019378000	0.232957000
C	2.014971000	-3.589664000	0.241010000
C	2.076677000	3.536682000	0.241302000
C	-3.563829000	-1.820636000	-1.120182000
C	3.304212000	-2.169076000	-1.102137000
C	0.218750000	3.965362000	-1.112427000
C	-2.693826000	-2.940873000	-1.394329000
C	3.868151000	-0.874942000	-1.386886000
C	-1.181361000	3.748543000	-1.387184000
C	-1.802264000	-2.743889000	-2.511197000
C	3.265905000	-0.201222000	-2.512449000
C	-1.465369000	2.901395000	-2.511470000
C	-3.573932000	-0.622325000	-1.941107000
C	2.295169000	-2.795224000	-1.930443000
C	1.258280000	3.381471000	-1.931295000
C	0.355864000	-2.375482000	-3.355591000
C	1.860653000	1.483512000	-3.347106000
C	-2.232555000	0.858988000	-3.366827000
C	-2.688196000	-0.461514000	-3.051161000
C	1.726146000	-2.109865000	-3.042936000
C	0.948642000	2.542236000	-3.045000000
C	-1.824176000	-1.558881000	-3.346275000
C	2.247343000	-0.812697000	-3.339854000
C	-0.429657000	2.339202000	-3.346342000
C	-0.783939000	-4.105721000	-0.243364000

C	3.983360000	1.395307000	-0.248298000
C	-3.107474000	2.671413000	-0.247223000
C	0.110118000	-3.874255000	-1.364959000
C	3.306407000	2.034802000	-1.365803000
C	-3.385511000	1.804676000	-1.373939000
C	-3.914701000	0.499981000	-1.109312000
C	1.505555000	-3.669459000	-1.104422000
C	2.404813000	3.128090000	-1.099024000
C	-0.436907000	-3.234626000	-2.515807000
C	3.006777000	1.228263000	-2.508061000
C	-2.578708000	1.972700000	-2.529052000
C	-0.499913000	-1.335996000	-3.858311000
C	1.392223000	0.223928000	-3.850319000
C	-0.899736000	1.080795000	-3.861590000
C	-0.003066000	-0.010461000	-4.079065000
C	-2.192752000	-3.709385000	-0.246508000
C	4.294605000	-0.050715000	-0.248996000
C	-2.094271000	3.715637000	-0.244421000
C	0.416510000	-2.357922000	3.350859000
C	1.810449000	1.539154000	3.347326000
C	-2.254331000	0.794696000	3.351004000
C	-0.963502000	-2.561132000	3.049866000
C	2.664516000	0.437944000	3.042756000
C	-1.732546000	2.089698000	3.050833000
C	-1.300706000	-3.418397000	1.929789000
C	3.541089000	0.579569000	1.905120000
C	-2.295023000	2.813586000	1.928730000
C	-1.867107000	-1.501978000	3.354496000
C	2.214972000	-0.875852000	3.358608000
C	-0.366417000	2.347921000	3.356114000
C	-1.399786000	-0.242267000	3.868043000
C	0.889086000	-1.099370000	3.870373000
C	0.489567000	1.319250000	3.872704000
C	-0.006460000	-0.008275000	4.086914000
N	0.145787000	0.230586000	-0.004426000
Tb	-1.113423000	-1.547259000	-0.015510000
Sc	2.083796000	0.124087000	-0.265247000
Sc	-0.706735000	1.988516000	0.159846000

$DySc_2N@C_{80}$

C	0.827070000	2.554839000	3.109441000
C	0.442936000	1.423108000	3.904970000
C	-0.982670000	1.107303000	4.012761000
C	1.368465000	0.314968000	3.889197000
C	2.081614000	2.615836000	2.386235000
C	3.723238000	1.261702000	1.158590000
C	2.965321000	1.504119000	2.368486000
C	2.613135000	0.367758000	3.151345000
C	0.913632000	-1.064759000	3.874843000
C	-0.488189000	-1.402872000	3.885608000
C	-1.444527000	-0.303048000	4.001804000
C	-0.855631000	-2.547187000	3.100417000
C	1.880902000	-1.841135000	3.131574000
C	2.231284000	-3.242492000	1.161591000
C	1.489811000	-2.973592000	2.353902000
C	0.112107000	-3.338584000	2.377823000
C	4.167385000	-0.051926000	0.730006000
C	3.656009000	-1.194434000	1.471197000
C	2.931407000	-0.959606000	2.694959000
C	3.282258000	-2.355278000	0.724053000
C	-2.103066000	-2.621012000	2.365464000
C	-2.978345000	-1.504679000	2.330646000
C	-2.612939000	-0.359042000	3.138269000
C	-3.723854000	-1.273159000	1.129709000
C	-1.903796000	-3.450448000	1.209936000
C	-1.893028000	-3.428037000	-1.241478000
C	-2.580376000	-3.163983000	-0.018677000
C	-3.529973000	-2.096221000	-0.033629000
C	1.588359000	-3.775587000	-0.005304000
C	0.185716000	-4.069253000	-0.009831000
C	-0.531733000	-3.883340000	1.212216000
C	-0.529058000	-3.879395000	-1.237298000
C	-4.041860000	0.056686000	0.685211000
C	-3.609646000	1.199705000	1.421168000
C	-2.901531000	0.962435000	2.654779000
C	-3.282090000	2.374561000	0.696447000
C	-4.061487000	0.060736000	-0.759754000
C	-3.037492000	0.997709000	-2.806869000
C	-3.668858000	1.220479000	-1.513054000
C	-3.284276000	2.375188000	-0.748457000
C	-2.100759000	-2.600047000	-2.413308000
C	-3.063593000	-1.527633000	-2.464355000
C	-3.751513000	-1.284252000	-1.217420000
C	-2.744148000	-0.363954000	-3.302885000
C	-2.239400000	3.259844000	1.150842000
C	-1.502619000	2.993255000	2.344128000
C	-1.889355000	1.852633000	3.149416000

C	-0.127601000	3.361611000	2.376671000
C	-1.589138000	3.808440000	-0.010698000
C	0.528447000	3.906303000	-1.228406000
C	-0.188018000	4.091873000	-0.006627000
C	0.526114000	3.908941000	1.223076000
C	-1.914724000	1.850646000	-3.156165000
C	-1.496049000	2.992667000	-2.368332000
C	-2.229913000	3.264230000	-1.177445000
C	-0.117186000	3.357069000	-2.393598000
C	1.894339000	3.455334000	-1.222775000
C	2.565011000	3.171066000	0.000320000
C	1.887431000	3.445015000	1.225426000
C	3.502330000	2.073914000	-0.002618000
C	2.101704000	2.631637000	-2.387789000
C	2.635432000	0.373823000	-3.142453000
C	2.988108000	1.515227000	-2.363363000
C	3.779476000	1.276706000	-1.170508000
C	-0.937061000	1.060314000	-3.851465000
C	1.401560000	0.312997000	-3.872031000
C	0.469197000	1.394154000	-3.815849000
C	0.852470000	2.567687000	-3.110080000
C	-0.834728000	-2.548117000	-3.120213000
C	0.946312000	-1.054346000	-3.865477000
C	-0.450103000	-1.378544000	-3.839115000
C	-1.419654000	-0.300331000	-3.921680000
C	2.239157000	-3.233164000	-1.166478000
C	1.902845000	-1.838231000	-3.130749000
C	1.502641000	-2.969472000	-2.362178000
C	0.123149000	-3.345912000	-2.401742000
C	4.239143000	-0.049024000	-0.746853000
C	2.961305000	-0.961052000	-2.676904000
C	3.738353000	-1.211053000	-1.490961000
C	3.305669000	-2.358052000	-0.728418000
N	0.177118000	0.014674000	0.192374000
Dy	-1.351784000	-0.081902000	-1.348894000
Sc	2.087332000	-0.167067000	-0.213543000
Sc	-0.375334000	0.166463000	2.070833000

$HoSc_2N@C_{80}$

C	-3.014605000	-1.253402000	2.515668000
C	2.571967000	-1.996943000	2.527251000
C	0.420976000	3.220076000	2.509858000
C	-3.319746000	-2.077337000	1.378471000
C	3.391408000	-1.835892000	1.371017000
C	-0.137263000	3.926998000	1.392457000
C	-2.498719000	-3.253836000	1.126991000
C	3.970699000	-0.533811000	1.098669000
C	-1.572776000	3.801674000	1.141779000
C	-3.878723000	-1.405401000	0.237718000
C	3.117215000	-2.668844000	0.245786000
C	0.726714000	4.057912000	0.244931000
C	-3.248331000	0.172596000	2.515281000
C	1.454058000	-2.910718000	2.518174000
C	1.776949000	2.710803000	2.512653000
C	-0.256171000	-3.943885000	1.093972000
C	3.530218000	1.754184000	1.084674000
C	-3.253911000	2.155997000	1.086043000
C	-3.740827000	0.825782000	1.355797000
C	1.139157000	-3.678724000	1.363851000
C	2.600446000	2.828148000	1.361043000
C	-4.096880000	0.022149000	0.232857000
C	2.016673000	-3.589281000	0.240863000
C	2.076363000	3.535289000	0.241010000
C	-3.558937000	-1.815631000	-1.119626000
C	3.304190000	-2.168394000	-1.102542000
C	0.218415000	3.962917000	-1.112554000
C	-2.689700000	-2.935343000	-1.393631000
C	3.865877000	-0.873694000	-1.386852000
C	-1.181115000	3.747353000	-1.387394000
C	-1.799405000	-2.739493000	-2.510574000
C	3.263063000	-0.200574000	-2.511803000
C	-1.465259000	2.901339000	-2.512144000
C	-3.573270000	-0.619296000	-1.941833000
C	2.296111000	-2.794724000	-1.931416000
C	1.258183000	3.381616000	-1.932381000
C	0.356870000	-2.374305000	-3.357836000
C	1.860440000	1.484231000	-3.349429000
C	-2.232725000	0.860445000	-3.368606000
C	-2.688106000	-0.459620000	-3.052461000
C	1.726938000	-2.109339000	-3.044506000
C	0.948587000	2.542841000	-3.046461000
C	-1.823068000	-1.556353000	-3.347155000
C	2.247078000	-0.812178000	-3.341685000
C	-0.429717000	2.339906000	-3.347833000
C	-0.781188000	-4.100190000	-0.243787000
C	3.974889000	1.392290000	-0.247751000

C	-3.108575000	2.671717000	-0.247397000
C	0.112210000	-3.870918000	-1.365469000
C	3.300948000	2.032660000	-1.365115000
C	-3.386783000	1.805972000	-1.374806000
C	-3.917035000	0.502315000	-1.110302000
C	1.507537000	-3.669003000	-1.104923000
C	2.404051000	3.128341000	-1.099665000
C	-0.434884000	-3.231650000	-2.516392000
C	3.003832000	1.228159000	-2.508301000
C	-2.578888000	1.973374000	-2.529826000
C	-0.499315000	-1.334697000	-3.860665000
C	1.392387000	0.224556000	-3.853199000
C	-0.899889000	1.081967000	-3.863712000
C	-0.002915000	-0.009310000	-4.081792000
C	-2.189382000	-3.704003000	-0.246633000
C	4.290715000	-0.050603000	-0.248582000
C	-2.093237000	3.713474000	-0.244711000
C	0.418024000	-2.356892000	3.352451000
C	1.811385000	1.539517000	3.349605000
C	-2.253950000	0.795863000	3.352831000
C	-0.961784000	-2.557943000	3.050939000
C	2.665327000	0.438256000	3.045128000
C	-1.731764000	2.089970000	3.052141000
C	-1.297346000	-3.411579000	1.928497000
C	3.541030000	0.579162000	1.906756000
C	-2.293755000	2.811294000	1.928301000
C	-1.866283000	-1.500173000	3.356876000
C	2.216272000	-0.875577000	3.361184000
C	-0.365779000	2.348346000	3.358116000
C	-1.399343000	-0.240985000	3.870678000
C	0.890338000	-1.098771000	3.872659000
C	0.490368000	1.319748000	3.874984000
C	-0.005656000	-0.007566000	4.089264000
N	0.128325000	0.204157000	-0.007861000
Ho	-1.119039000	-1.562934000	-0.017950000
Sc	2.076515000	0.101470000	-0.251876000
Sc	-0.716705000	1.974949000	0.149342000

ErSc$_2$N@C$_{80}$

C	-3.013898000	-1.252155000	2.516467000
C	2.572618000	-1.997090000	2.528225000
C	0.421233000	3.219541000	2.510184000
C	-3.317468000	-2.074763000	1.378155000
C	3.391339000	-1.836175000	1.371395000
C	-0.136846000	3.924536000	1.391764000
C	-2.496748000	-3.250841000	1.126597000
C	3.969225000	-0.533867000	1.098838000
C	-1.571766000	3.799459000	1.141148000
C	-3.876549000	-1.403166000	0.237629000
C	3.117972000	-2.669220000	0.245860000
C	0.726743000	4.056056000	0.244682000
C	-3.248396000	0.173374000	2.515991000
C	1.454862000	-2.910713000	2.519046000
C	1.777086000	2.710863000	2.513169000
C	-0.254722000	-3.940467000	1.093381000
C	3.528641000	1.753751000	1.084789000
C	-3.254216000	2.156007000	1.086257000
C	-3.741592000	0.826311000	1.356317000
C	1.140483000	-3.678036000	1.364060000
C	2.600134000	2.828125000	1.361237000
C	-4.097503000	0.023469000	0.232748000
C	2.017898000	-3.589903000	0.240847000
C	2.076399000	3.534895000	0.240934000
C	-3.556570000	-1.813200000	-1.119372000
C	3.304252000	-2.168428000	-1.102641000
C	0.218570000	3.961428000	-1.112482000
C	-2.687420000	-2.932378000	-1.393188000
C	3.864709000	-0.873466000	-1.386802000
C	-1.180487000	3.745700000	-1.387123000
C	-1.797779000	-2.737298000	-2.510164000
C	3.262240000	-0.200411000	-2.511770000
C	-1.465088000	2.901016000	-2.512479000
C	-3.573239000	-0.617886000	-1.942374000
C	2.296749000	-2.794982000	-1.931975000
C	1.258361000	3.381629000	-1.932812000
C	0.357519000	-2.373835000	-3.358915000
C	1.860396000	1.484355000	-3.350112000
C	-2.232793000	0.861115000	-3.369571000
C	-2.688065000	-0.458702000	-3.053158000
C	1.727561000	-2.109360000	-3.045325000
C	0.948664000	2.543001000	-3.046947000
C	-1.822396000	-1.555157000	-3.347519000
C	2.247124000	-0.812066000	-3.342456000
C	-0.429602000	2.340032000	-3.348364000
C	-0.779811000	-4.097322000	-0.244002000
C	3.971733000	1.391323000	-0.247548000

C	-3.108665000	2.671355000	-0.247484000
C	0.113342000	-3.869457000	-1.365705000
C	3.299284000	2.032038000	-1.364908000
C	-3.387501000	1.806538000	-1.375445000
C	-3.918396000	0.503421000	-1.110967000
C	1.508721000	-3.669359000	-1.105245000
C	2.403907000	3.128351000	-1.099873000
C	-0.433779000	-3.230196000	-2.516650000
C	3.002996000	1.228073000	-2.508401000
C	-2.579047000	1.973729000	-2.530410000
C	-0.498900000	-1.334140000	-3.861876000
C	1.392513000	0.224754000	-3.854209000
C	-0.899858000	1.082345000	-3.864601000
C	-0.002782000	-0.008889000	-4.082908000
C	-2.187559000	-3.701082000	-0.246629000
C	4.288093000	-0.050607000	-0.248420000
C	-2.092291000	3.711761000	-0.244678000
C	0.418708000	-2.356485000	3.353265000
C	1.811642000	1.539748000	3.350460000
C	-2.254083000	0.796472000	3.353969000
C	-0.961038000	-2.556425000	3.051481000
C	2.665359000	0.438361000	3.045918000
C	-1.731640000	2.090119000	3.052782000
C	-1.295831000	-3.408225000	1.927935000
C	3.540076000	0.579028000	1.907043000
C	-2.293473000	2.810589000	1.928321000
C	-1.866017000	-1.499303000	3.358116000
C	2.216766000	-0.875459000	3.362095000
C	-0.365614000	2.348418000	3.358663000
C	-1.399272000	-0.240295000	3.872079000
C	0.890803000	-1.098481000	3.873482000
C	0.490625000	1.319951000	3.875757000
C	-0.005394000	-0.007231000	4.090178000
N	0.119412000	0.191197000	-0.008676000
Er	-1.121756000	-1.569913000	-0.017944000
Sc	2.072252000	0.095949000	-0.251284000
Sc	-0.721165000	1.968256000	0.149280000

$TmSc_2N@C_{80}$

C	-3.013091000	-1.251082000	2.517304000
C	2.573159000	-1.995951000	2.528781000
C	0.421721000	3.219468000	2.511512000
C	-3.315373000	-2.072324000	1.378103000
C	3.393072000	-1.834913000	1.372276000
C	-0.136135000	3.920692000	1.390857000
C	-2.494785000	-3.247861000	1.126237000
C	3.974996000	-0.534016000	1.101436000
C	-1.569936000	3.795330000	1.139751000
C	-3.875202000	-1.401507000	0.237630000
C	3.117586000	-2.666337000	0.245428000
C	0.726634000	4.054348000	0.244283000
C	-3.247801000	0.174188000	2.516556000
C	1.455552000	-2.909460000	2.519405000
C	1.777021000	2.710853000	2.513780000
C	-0.253520000	-3.936954000	1.092847000
C	3.525904000	1.752453000	1.085118000
C	-3.254108000	2.155652000	1.086182000
C	-3.741720000	0.826530000	1.356755000
C	1.141437000	-3.676147000	1.364040000
C	2.599219000	2.827785000	1.361199000
C	-4.097939000	0.024398000	0.232772000
C	2.017767000	-3.587500000	0.240406000
C	2.076523000	3.534850000	0.240436000
C	-3.555287000	-1.811508000	-1.119433000
C	3.304898000	-2.167510000	-1.103677000
C	0.218093000	3.961282000	-1.112878000
C	-2.686280000	-2.930511000	-1.393350000
C	3.864434000	-0.872022000	-1.387455000
C	-1.180979000	3.746763000	-1.387742000
C	-1.796915000	-2.735546000	-2.510314000
C	3.259954000	-0.199522000	-2.511084000
C	-1.465120000	2.901246000	-2.512616000
C	-3.573245000	-0.616782000	-1.942778000
C	2.297428000	-2.794004000	-1.932933000
C	1.258159000	3.382342000	-1.933594000
C	0.357814000	-2.373058000	-3.360343000
C	1.860432000	1.485323000	-3.352273000
C	-2.232950000	0.861776000	-3.370373000
C	-2.688200000	-0.457937000	-3.053810000
C	1.727810000	-2.108562000	-3.046543000
C	0.948598000	2.543618000	-3.047878000
C	-1.822128000	-1.553998000	-3.348214000
C	2.246847000	-0.811426000	-3.343880000
C	-0.429741000	2.340499000	-3.349056000
C	-0.778467000	-4.093928000	-0.244384000
C	3.964089000	1.388410000	-0.247325000

C	-3.109340000	2.671611000	-0.247609000
C	0.114143000	-3.867279000	-1.366303000
C	3.294083000	2.030425000	-1.364459000
C	-3.387954000	1.806925000	-1.375641000
C	-3.919176000	0.504204000	-1.111204000
C	1.509443000	-3.667793000	-1.105708000
C	2.403633000	3.129776000	-1.100657000
C	-0.433120000	-3.228884000	-2.517440000
C	3.000581000	1.228521000	-2.508943000
C	-2.579077000	1.973867000	-2.530554000
C	-0.498775000	-1.333377000	-3.863236000
C	1.392658000	0.225384000	-3.856473000
C	-0.900032000	1.083097000	-3.865723000
C	-0.002673000	-0.008112000	-4.084593000
C	-2.186190000	-3.698553000	-0.246788000
C	4.287822000	-0.050266000	-0.248366000
C	-2.092196000	3.711304000	-0.245041000
C	0.419550000	-2.355618000	3.354078000
C	1.812282000	1.539935000	3.351645000
C	-2.253597000	0.796970000	3.354902000
C	-0.960233000	-2.554860000	3.052236000
C	2.666349000	0.438798000	3.047565000
C	-1.730839000	2.090155000	3.053564000
C	-1.294252000	-3.404822000	1.927350000
C	3.541990000	0.578817000	1.908744000
C	-2.292028000	2.808470000	1.927641000
C	-1.865716000	-1.498613000	3.359689000
C	2.217574000	-0.874991000	3.363898000
C	-0.365184000	2.349042000	3.360385000
C	-1.399041000	-0.239722000	3.873698000
C	0.891616000	-1.097953000	3.875105000
C	0.491099000	1.320371000	3.877299000
C	-0.004945000	-0.006687000	4.091855000
N	0.112498000	0.178408000	-0.009858000
Tm	-1.127246000	-1.574406000	-0.021364000
Sc	2.070975000	0.065930000	-0.230004000
Sc	-0.724905000	1.962484000	0.136282000

$LuSc_2N@C_{80}$

C	-3.013521000	-1.250367000	2.519673000
C	2.573257000	-1.996111000	2.529320000
C	0.421757000	3.219270000	2.511921000
C	-3.313526000	-2.069592000	1.378744000
C	3.392785000	-1.835289000	1.372445000
C	-0.136143000	3.919311000	1.390641000
C	-2.493821000	-3.246112000	1.126940000
C	3.974127000	-0.534248000	1.101446000
C	-1.569658000	3.794128000	1.139554000
C	-3.874126000	-1.398700000	0.237811000
C	3.118172000	-2.667161000	0.245505000
C	0.726405000	4.053086000	0.244197000
C	-3.248409000	0.174801000	2.517355000
C	1.456190000	-2.910179000	2.520692000
C	1.776953000	2.710850000	2.514118000
C	-0.251776000	-3.934420000	1.092835000
C	3.524679000	1.751975000	1.085045000
C	-3.254402000	2.155537000	1.086419000
C	-3.743237000	0.827039000	1.357429000
C	1.142995000	-3.676336000	1.364515000
C	2.598974000	2.827764000	1.361376000
C	-4.099744000	0.026016000	0.232599000
C	2.019329000	-3.589183000	0.240382000
C	2.076289000	3.534345000	0.240373000
C	-3.553216000	-1.808280000	-1.119787000
C	3.304825000	-2.167514000	-1.103714000
C	0.217964000	3.960319000	-1.112922000
C	-2.683592000	-2.927091000	-1.393689000
C	3.864093000	-0.872046000	-1.387473000
C	-1.180809000	3.745671000	-1.387604000
C	-1.795608000	-2.734369000	-2.511989000
C	3.259482000	-0.199490000	-2.510989000
C	-1.465062000	2.900741000	-2.512663000
C	-3.574370000	-0.615181000	-1.944166000
C	2.297859000	-2.794344000	-1.933430000
C	1.258159000	3.382469000	-1.934030000
C	0.358369000	-2.373614000	-3.362577000
C	1.860473000	1.485370000	-3.352738000
C	-2.233162000	0.862090000	-3.371203000
C	-2.689089000	-0.457524000	-3.055466000
C	1.728146000	-2.108913000	-3.047367000
C	0.948567000	2.543692000	-3.048218000
C	-1.822187000	-1.553370000	-3.350026000
C	2.246683000	-0.811388000	-3.344084000
C	-0.429709000	2.340440000	-3.349351000
C	-0.776840000	-4.092722000	-0.244846000
C	3.962337000	1.387709000	-0.247274000

C	-3.109539000	2.671303000	-0.247616000
C	0.115711000	-3.866648000	-1.367209000
C	3.293156000	2.030077000	-1.364442000
C	-3.388887000	1.807168000	-1.376134000
C	-3.921985000	0.505504000	-1.112237000
C	1.510951000	-3.668960000	-1.106113000
C	2.403510000	3.129814000	-1.100909000
C	-0.431806000	-3.229321000	-2.519643000
C	3.000289000	1.228516000	-2.509204000
C	-2.579210000	1.973818000	-2.530827000
C	-0.498598000	-1.333308000	-3.865019000
C	1.392723000	0.225458000	-3.856966000
C	-0.900097000	1.083235000	-3.866426000
C	-0.002614000	-0.008037000	-4.085371000
C	-2.184261000	-3.695816000	-0.246825000
C	4.286694000	-0.050463000	-0.248260000
C	-2.091855000	3.710201000	-0.244954000
C	0.419963000	-2.356038000	3.355431000
C	1.812331000	1.540096000	3.352164000
C	-2.253812000	0.797288000	3.355699000
C	-0.960285000	-2.554776000	3.054661000
C	2.666165000	0.438850000	3.047896000
C	-1.730781000	2.090155000	3.054162000
C	-1.293222000	-3.403117000	1.928509000
C	3.541201000	0.578584000	1.908750000
C	-2.291942000	2.807895000	1.927760000
C	-1.866593000	-1.498884000	3.362839000
C	2.217552000	-0.874900000	3.364226000
C	-0.365145000	2.349263000	3.361111000
C	-1.399415000	-0.239641000	3.875275000
C	0.891674000	-1.097920000	3.875581000
C	0.491098000	1.320488000	3.877783000
C	-0.005055000	-0.006637000	4.092382000
N	0.106564000	0.169678000	-0.012355000
Lu	-1.122728000	-1.573171000	-0.017416000
Sc	2.069629000	0.061541000	-0.231244000
Sc	-0.728243000	1.959621000	0.136344000
